# Correction: Reconstructing prehistoric lifeways using multi-Isotope analyses of human enamel, dentine, and bone from Legaire Sur, Spain

**DOI:** 10.1371/journal.pone.0321333

**Published:** 2025-04-01

**Authors:** The PLOS One Staff

## Notice of Republication

This article was republished on March 3, 2025, to correct an error in the data availability statement and to include the Inclusivity in Global Research Questionnaire (S2 File), which was inadvertently missed during the publication process. The publisher apologizes for these errors. Please download the article again to view the correct version. The originally published, uncorrected article and the republished, corrected article are provided here for reference.

## Supporting Information

S1 File Originally published, uncorrected article. (PDF)

S2 File Republished, corrected article. (PDF)
