# Supplementary material for: Reconstructing prehistoric lifeways using multi-Isotope analyses of human enamel, dentine, and bone from Legaire Sur, Spain
Source: PLoS One. 2025 Jan 22;20(1):e0316387. doi: 10.1371/journal.pone.0316387 (PMC11753681; doi:10.1371/journal.pone.0316387)
Supplement: S1 File — (DOCX) [file pone.0316387.s001.docx]

# **SUPPORTING INFORMATION:** Collagen Extraction & Faunal Baseline.

Jacob I. Griffith^1^, Hannah F. James^1^, Javier Ordoño^2^, Teresa Fernández-Crespo^3,4^, Carina T. Gerritzen^1^, Christina Cheung^1,5^, Rachèl Spros^1,6^, Philippe Claeys^1^, Steven Goderis^1^, Barbara Veselka^1^, Christophe Snoeck^1^

^1^Archaeology, Environmental Changes and Geo-Chemistry, Vrije Universiteit Brussel, Belgium.

^2^Department of Archaeology and New Technologies, Arkikus, Spain.

^3^Departamento de Prehistoria, Arqueología, Antropología Social y Ciencias y Técnicas Historiográficas, Universidad de Valladolid, Spain.

^4^Research Laboratory for Archaeology and the History of Art, School of Archaeology, University of Oxford, UK.

^5^Department of Anthropology, Chinese University of Hong Kong, Hong Kong.

^6^Social History of Capitalism, Department of History, Archaeology, Arts, Philosophy and Ethics, Vrije Universiteit Brussel, Belgium.

# SI.SECTION 1: Collagen Extraction Protocol for Dentine Increments & Bone Collagen

All bone samples were cut and prepared in the AMGC labs of the VUB. Before sampling, the surface of each bone sample was cleaned using a 1mm cylindrical bur attached to the handrill, at a low RPM, in order to reduce contamination of samples. For each bone sample, between 400-600mg of material was cut using an attached diamond wheel saw. Each sample was placed into labelled glass 15ml test tubes, which were then filled with 10ml (2/3 full) of 0.5 molar hydrochloric acid. The same process was applied to the slices of teeth for later dentine increment extraction. 0.5m HCl was created by diluting 6M HCl (83ml) with deionised water (917ml) (1:11 6M HCl:H_2_O). The samples were then covered and refrigerated (approx. 4^o^C), to prevent microbial contamination of the organic samples, which could cause discrepancies within the resulting δ^13^C and δ^15^N values [85].

Samples were kept in 0.5M HCl for 48 hours before being reviewed; when the HCl was drained and the sampled extracted using sterile tweezers onto a sterilised 40-mesh sieve. Inspecting the structural integrity of the sample revealed whether complete demineralisation had occurred. If a sample required further demineralisation, the process was repeated, with the dentine being submerged into fresh 0.5m HCl and reviewed after 48 hours. This cycle was repeated until complete demineralisation had occurred, with most samples taking three 0.5m HCl changes.

On full demineralisation, the 0.5m HCl was drained through a sterilised 40-mesh sieve and the sample removed from the test tube using sterilised tweezers. The samples and test tube were washed thoroughly with deionised water to neutralise any residual 0.5M HCl which could cause unwanted degradation of the collagen sample. Following [86] samples were then refrigerated overnight (>12 hours) in deionised water in order to guarantee complete neutralisation of the demineralised bone and dentine. After, the tooth slices were seperated from the collagen extraction process for incremental sampling.

For the bone samples, the deionised water was drained and replaced with 10 ml pH3 H2O; created by diluting HCl (2ml 0.5M HCl) with deionised water (9,998ml H2O) (1:449 0.5m HCl:H2O). The test tubes were sealed and placed onto a preheated hotblock for 48 hours, at 75oC, to enable the gelatinisation process.

After 48 hours the samples were removed from the hotblock and allowed to cool (+/-30 minutes). They were then filtered using an 8-μm EZEE filter, separating the gelatinised collagen solution from the remaining insoluble residue.

This solution was transferred into labelled 3.5ml plastic test tubes and tightly sealed with Parafilm® ‘M’ Barrier Film. The Parafilm® was then pierced, allowing gas to escape during the freeze-drying process and placed into a freezer at -20^o^C for <12 hours. Following [88], the samples were stored at a slight angle, increasing the surface area of the frozen solution, to assist the later freeze-drying process.

To isolate the collagen from the acquired gelatinised solutions, freeze-drying (lyophilisation), was required to precipitate the samples. Frozen samples were transferred from the freezer into the freeze-dryer quickly to prevent any defrosting before dehydration. Samples were kept at -40oC for 72 hours, during which time all liquid was removed through the sublimation of ice [88], producing pure collagen. Successfully freeze-dried samples were dry, with a white to light brown colouration. Immediately after their transfer from the freeze-dryer, Parafilm® was removed and samples were re-sealed, to prevent collagen rehydration through atmospheric water vapour. Samples with a crusted, dark brown residue, required refreshing, freezing and freeze-drying [89]. This was achieved by hydrating the samples with 2ml of deionised water and returning them to the hotblock for an hour, in order to encourage further dissolution [86]. After re-freezing (<12 Hours) and re-freeze-drying (72 hours), if the samples did not produce viable collagen, they were disposed of.

1.0mg ±0.2 of each collagen specimen selected for analysis was placed into a 5x3.5mm SerCon tin capsule, where it was folded into a <2.5mm sphere, placed into a tray and vacuum stored for later combustion and mass spectrometry. This process was performed quickly and in duplicate for each specimen due to the hygroscopic nature of dried collagen.

## References

85. Leatherdale A. Interpreting stable carbon and nitrogen isotope ratios in archaeological remains: An overview of the processes influencing the δ13C and δ15N values of type I collagen. The University of Western Ontario Journal of Anthropology. 2013 Apr 28;21(1).

86. Jones JR. Land and sea: understanding diet and economies through time in the North Atlantic Islands (Doctoral dissertation, Cardiff University).

87. Towers JR. An isotopic investigation into calving seasonality, diet and dairying in British Prehistoric cattle. Reconstructing animal husbandry at a sub-annual resolution using multi-isotope analysis and intra-tooth sampling (Doctoral dissertation, University of Bradford).

88. Rey L. Glimpses Into The Realm Of Freeze-Drying: Fundamental Issues. Drugs And The Pharmaceutical Sciences. 2004 21;137:1-32.

89. Szpak P, Metcalfe JZ, Macdonald RA. Best practices for calibrating and reporting stable isotope measurements in archaeology. Journal of Archaeological Science: Reports. 2017 1;13:609-16.

# SI.Table 1: Faunal Baseline Samples

| **Site Name** | **Period** | **Species** | **Element** | **Publication** | **δ^13^C (‰)** | **δ^15^N (‰)** | **C:N** |
| --- | --- | --- | --- | --- | --- | --- | --- |
| Santimamiñe | Neolithic | Ovis aries/Capra hircus | Unspecified | Sarasketa-Gartzia *et al.* 2018 | -20.6 | 3.3 | 3.4 |
| Santimamiñe | Mesolithic | Ovis aries/Capra hircus | Unspecified | Sarasketa-Gartzia *et al.* 2018 | -21.4 | 5.8 | 3.3 |
| Santimamiñe | Neolithic | Capra pyrenaica | Unspecified | Sarasketa-Gartzia *et al.* 2018 | -20.1 | 2.7 | 3.3 |
| Santimamiñe | Mesolithic | Capra pyrenaica | Unspecified | Sarasketa-Gartzia *et al.* 2018 | -20 | 2.6 | 3.3 |
| Santimamiñe | Neolithic | Cervus elaphus | Unspecified | Sarasketa-Gartzia *et al.* 2018 | -21.6 | 2.7 | 3.2 |
| Santimamiñe | Chalcolithic/Bronze Age | Ovis aries/Capra hircus | Unspecified | Sarasketa-Gartzia *et al.* 2018 | -19.9 | 3.2 | 3.3 |
| Santimamiñe | Mesolithic | Sus Scrofa | Unspecified | Sarasketa-Gartzia *et al.* 2018 | -20.2 | 3.9 | 3.3 |
| Santimamiñe | Mesolithic | Cervus elaphus | Unspecified | Sarasketa-Gartzia *et al.* 2018 | -21.1 | 2.7 | 3.3 |
| Pico Ramos | Chalcolithic | Ovis aries/Capra hircus | Unspecified | Sarasketa-Gartzia *et al.* 2018 | -20.3 | 3.5 | 3.3 |
| Pico Ramos | Chalcolithic | Sus Scrofa | Unspecified | Sarasketa-Gartzia *et al.* 2018 | -21.1 | 4.9 | 3.3 |
| Pico Ramos | Chalcolithic | Cervus elaphus | Unspecified | Sarasketa-Gartzia *et al.* 2018 | -20.6 | 3.3 | 3.5 |
| Pico Ramos | Chalcolithic | Bos taurus | Unspecified | Sarasketa-Gartzia *et al.* 2018 | -21.9 | 4.8 | 3.4 |
| Los Husos I | Late Neolithic/Chalcolithic | Bos taurus | Humerus | Fernandez-Crespo & Schulting 2017 | -20.6 | 4.1 | 3.4 |
| Los Husos I | Late Neolithic/Chalcolithic | Bos taurus | Mandible | Fernandez-Crespo & Schulting 2017 | -21.1 | 4.1 | 3.4 |
| Los Husos I | Late Neolithic/Chalcolithic | Bos taurus | Tibia | Fernandez-Crespo & Schulting 2017 | -20.7 | 5.7 | 3.3 |
| Los Husos I | Late Neolithic/Chalcolithic | Ovis aries/Capra hircus | Femur | Fernandez-Crespo & Schulting 2017 | -20.5 | 3.9 | 3.3 |
| Los Husos I | Late Neolithic/Chalcolithic | Ovis aries/Capra hircus | Femur | Fernandez-Crespo & Schulting 2017 | -20.4 | 6.6 | 3.3 |
| Los Husos I | Late Neolithic/Chalcolithic | Ovis aries/Capra hircus | Mandible | Fernandez-Crespo & Schulting 2017 | -21.1 | 3.3 | 3.3 |
| Los Husos I | Late Neolithic/Chalcolithic | Ovis aries/Capra hircus | Mandible | Fernandez-Crespo & Schulting 2017 | -20.7 | 5.5 | 3.3 |
| Los Husos I | Late Neolithic/Chalcolithic | Ovis aries/Capra hircus | Mandible | Fernandez-Crespo & Schulting 2017 | -20.9 | 6.2 | 3.3 |
| Los Husos I | Late Neolithic/Chalcolithic | Ovis aries/Capra hircus | Mandible | Fernandez-Crespo & Schulting 2017 | -20.9 | 4.4 | 3.4 |
| Los Husos I | Late Neolithic/Chalcolithic | Ovis aries/Capra hircus | Mandible | Fernandez-Crespo & Schulting 2017 | -20.4 | 5.1 | 3.3 |
| Los Husos I | Late Neolithic/Chalcolithic | Ovis aries/Capra hircus | Mandible | Fernandez-Crespo & Schulting 2017 | -20.9 | 4.7 | 3.4 |
| Los Husos I | Late Neolithic/Chalcolithic | Cervus elaphus | Femur | Fernandez-Crespo & Schulting 2017 | -19.8 | 6.4 | 3.3 |
| Los Husos I | Late Neolithic/Chalcolithic | Cervus elaphus | Mandible | Fernandez-Crespo & Schulting 2017 | -21.3 | 3.7 | 3.5 |
| Los Husos I | Late Neolithic/Chalcolithic | Cervus elaphus | Mandible | Fernandez-Crespo & Schulting 2017 | -20.6 | 7.2 | 3.5 |
| Los Husos I | Late Neolithic/Chalcolithic | Sus domesticus | Mandible | Fernandez-Crespo & Schulting 2017 | -20.8 | 5 | 3.5 |
| Los Husos I | Late Neolithic/Chalcolithic | Sus domesticus | Mandible | Fernandez-Crespo & Schulting 2017 | -21.1 | 8.7 | 3.3 |
| Los Husos I | Late Neolithic/Chalcolithic | Sus domesticus | Mandible | Fernandez-Crespo & Schulting 2017 | -20.3 | 5.6 | 3.4 |
| Los Husos I | Late Neolithic/Chalcolithic | Sus domesticus | Mandible | Fernandez-Crespo & Schulting 2017 | -19.7 | 2.6 | 3.4 |
| Peña Larga | Late Neolithic/Chalcolithic | Bos taurus | Mandible | Fernandez-Crespo & Schulting 2017 | -21.4 | 3.3 | 3.3 |
| Peña Larga | Late Neolithic/Chalcolithic | Ovis aries/Capra hircus | Humerus | Fernandez-Crespo & Schulting 2017 | -20.3 | 3.8 | 3.4 |
| Peña Larga | Late Neolithic/Chalcolithic | Cervus elaphus | Carpus | Fernandez-Crespo & Schulting 2017 | -20.5 | 3.6 | 3.3 |
| Peña Larga | Late Neolithic/Chalcolithic | Sus scrofa | Radius | Fernandez-Crespo & Schulting 2017 | -20.7 | 6.7 | 3.3 |
| El Sotillo | Late Neolithic/Chalcolithic | Bos taurus | Humerus | Fernandez-Crespo & Schulting 2017 | -20.9 | 6.1 | 3.5 |
| Alto de la Huesera | Late Neolithic/Chalcolithic | Bos taurus | Femur | Fernandez-Crespo & Schulting 2017 | -20.5 | 4.3 | 3.2 |
| Alto de la Huesera | Late Neolithic/Chalcolithic | Ovis aries/Capra hircus | Metapodial | Fernandez-Crespo & Schulting 2017 | -20.2 | 4.9 | 3.3 |
| Chabola de la Hechicera | Late Neolithic/Chalcolithic | Bos taurus | Rib | Fernandez-Crespo & Schulting 2017 | -20.2 | 5.9 | 3.2 |
| Chabola de la Hechicera | Late Neolithic/Chalcolithic | Ovis aries/Capra hircus | Humerus | Fernandez-Crespo & Schulting 2017 | -20.5 | 7.8 | 3.2 |
| Chabola de la Hechicera | Late Neolithic/Chalcolithic | Ovis aries/Capra hircus | Humerus | Fernandez-Crespo & Schulting 2017 | -20.6 | 4.8 | 3.2 |
| Chabola de la Hechicera | Late Neolithic/Chalcolithic | Sus domesticus | Mandible | Fernandez-Crespo & Schulting 2017 | -20.6 | 6.1 | 3.3 |
