# Supplementary material for: Correction: Reconstructing prehistoric lifeways using multi-Isotope analyses of human enamel, dentine, and bone from Legaire Sur, Spain
Source: PLoS One. 2025 Apr 1;20(4):e0321333. doi: 10.1371/journal.pone.0321333 (PMC11960974; doi:10.1371/journal.pone.0321333)
Supplement: S2 File — (PDF) [file pone.0321333.s002.pdf]

PLOS ONE

RESEARCH ARTICLE

Reconstructing prehistoric lifeways using multi-Isotope analyses of human enamel, dentine, and bone from Legaire Sur, Spain

Jacob I. Griffith[1]*, Hannah F. James[1], Javier Ordoño[2], Teresa Fernández-Crespo[3,4], Carina T. Gerritzen[1], Christina Cheung[1,5], Rachèl Spros[1,6], Philippe Claeys[1], Steven Goderis[1], Barbara Veselka[1], Christophe Snoeck[1]

1 Archaeology, Environmental Changes and Geo-Chemistry, Vrije Universiteit Brussel, Belgium, **2** Department of Archaeology and New Technologies, Arkikus, Spain, **3** Departamento de Prehistoria, Arqueología, Antropología Social y Ciencias y Técnicas Historiográficas, Universidad de Valladolid, Spain, **4** Research Laboratory for Archaeology and the History of Art, School of Archaeology, University of Oxford, United Kingdom, **5** Department of Anthropology, Chinese University of Hong Kong, Hong Kong, **6** Social History of Capitalism, Department of History, Archaeology, Arts, Philosophy and Ethics, Vrije Universiteit Brussel, Belgium

* jacob.griffith@vub.be

Check for updates

OPEN ACCESS

Citation: Griffith JI, James HF, Ordoño J, Fernández-Crespo T, Gerritzen CT, Cheung C, et al. (2025) Reconstructing prehistoric lifeways using multi-Isotope analyses of human enamel, dentine, and bone from Legaire Sur, Spain. PLoS ONE 20(1): e0316387. https://doi.org/10.1371/journal.pone.0316387

Editor: Luca Bondioli, University of Padova: Universita degli Studi di Padova, ITALY

Received: June 13, 2024

Accepted: December 10, 2024

Published: January 22, 2025

Copyright: © 2025 Griffith et al. This is an open access article distributed under the terms of the Creative Commons Attribution License, which permits unrestricted use, distribution, and reproduction in any medium, provided the original author and source are credited.

Data Availability Statement: The data supporting this study are available in the IsoArcH data repository (DOI: https://doi.org/10.48530/isoarch.2024.008).

Funding: Jacob I. Griffiths's personal Fonds Wetenschappelijk Onderzoek (FWO) funded doctoral fellowship titled: The Truth in the Tooth: Reconstructing and Comparing Human Life Histories in the Mesolithic and Neolithic of Europe (Scholarship Number: FWOTM1129). Funding also

Abstract

Megalithism has been repetitively tied to specialised herding economies in Iberia, particularly in the mountainous areas of the Basque Country. Legaire Sur, in the uplands of Álava region, is a recently excavated passage tomb (megalithic monument) that held a minimum number of 25 individuals. This study analysed the carbon, nitrogen, oxygen, and strontium isotope ratios of 18 individuals, in a multi-tissue sampling study (successional tooth enamel sampling, incremental dentine sampling, and bulk bone collagen sampling). The results provide a high-resolution reconstruction of individual mobility, weaning, and dietary lifeways of those inhumed at the site. Oxygen and strontium isotope analysis suggest all individuals come from a similar, likely local, geological region, aside from one biological female who presents a notably different geographical birthplace, weaning, and dietary life history than the rest of the burial population. Comparisons to other nearby megalithic sites ($\sim$35km as the crow flies), located in a valley area, reveal that, whilst sharing the same mortuary practices, these individuals held notably different lifeways. They highlight notably earlier ages of cessation of nursing ($\leq$2 years at Legaire Sur vs. $\geq$4 years in other megalithic tombs), and a greater dependence on pastoralism than previously observed in lowland megalithic graves. The results from Legaire Sur reveal the complexity of the Late Neolithic-Chalcolithic transition in north-central Iberia, categorising yet another separate socio-economic group with distinctive lifeways inhabiting the region.

Introduction

The Late Neolithic (ca. 3600–2900 BCE) to Chalcolithic (ca. 2900–2200 BCE) periods [1, 2] in Iberia, were a time characterised by a substantial population increase, the development of

by the ERC Starting Grant LUMIERE (Landscape Use and Mobility In EuRope – Bridging the gap between cremation and inhumation), funded by European Union's Horizon 2020 research and innovation programme under grant agreement number 948913 The funders had no role in study design, data collection and analysis, decision to publish, or preparation of the manuscript.

Competing interests: The authors have declared that no competing interests exist

metallurgical working of copper and gold, and settlement changing to walled ditch enclosures [3]. Other notable changes include the first evidence of intensive cereal production and ceramic technology refinement [1]. However, in north-central Iberia, much of this material evidence is relatively minimal; with very few metal elements being found and very few settlement sites known until the beginning of the Bell Beaker phenomenon (ca. 2500 BCE) [2].

Population growth in this region is seen however, by the increase and diversification of burial sites and funerary practices, including caves, rockshelters and megalithic tombs [3], occurring simultaneously during the Late Neolithic/Early Chalcolithic [4], and a notable increase in conflict and interpersonal violence [5–8]. Regardless of geographical burial location, individuals were deposited collectively, sharing the same mortuary space and grave goods. The cumulative use of the graves usually leads to commingled and fragmentary skeletal assemblages, although depositions are mainly primary burials. The social and economic shifts associated with the Late Neolithic-Chalcolithic transition are often comparatively difficult to interpret in the north-central region of Spain due to the lack of material culture. Bioarchaeological methods, such as isotope analyses, provide a unique opportunity to reconstruct the changing life histories and population dynamics in the region.

Over the past three decades, the use of isotope analyses in archaeological research has transformed from a highly specialised technique into a tool almost as common as the humble trowel. A natural progression and refinement of isotopic techniques has occurred, with new isotopic systems (δ^{88}Sr: [9–11]; $\delta^{42/44}$Ca: [12], δ^{66}Zn: [13], sampling techniques (e.g., [14–16]), and statistical analytical frameworks (e.g., [17, 18]) being developed and updated. The appeal of isotope analyses stems from their ability to reveal the direct actions of an individual, and a community, made within their lifetime.

Through the ingestion of food and drink, chemical elements are incorporated into the body tissues forming at that time (e.g., bone, tooth, hair, etc.; [19]), following the premise "you are what you eat" [20]. Carbon (δ^{13}C) and nitrogen (δ^{15}N) isotope ratios measured in the organic component of the skeletal matrix (i.e., in tooth dentine and bone collagen) [21] are useful in recreating the dietary choices of past individuals. Carbon (δ^{13}C), oxygen (δ^{18}O) and strontium isotope ratios (^{87}Sr/^{86}Sr, δ^{88}Sr, as well as strontium elemental concentrations [Sr]) can be measured in the mineral component of human remains (e.g., in tooth enamel; [19]). δ^{18}O and ^{87}Sr/^{86}Sr values can determine an individual's geographical origin, and, when compared to an individual's burial location, determine how the individual may have moved across their lifetime [22]. δ^{13}C, δ^{88}Sr and [Sr] can also infer dietary differences within a population [9, 11, 23, 24].

Isotope analyses have been instrumental in reconstructing lifeways in Late Neolithic-Chalcolithic north-central Iberia [4, 25–29]. Fernández-Crespo and Schulting [4] analysed δ^{13}C and δ^{15}N values from the bone collagen of 166 individuals from 4 passage tombs and 3 cave sites, located $\sim$35km from Legaire Sur. Notable adult diet differences were observed between the two mortuary populations, with the difference being linked to distinct economic specialisation or social status. Fernández-Crespo *et al.* [27] further isotopically analysed 32 individuals from six of these funerary sites. The results indicated a notable difference in infant- and child-rearing practices, in subsistence strategies, and in landscape use between burial locations. This led to a conclusion of a 'them and us' theory; with the two distinct cultural groups occupying a restricted region, holding different lifeways and presenting different mortuary practices [27], indicating a potential cause of the observed interpersonal violence in the region [8].

Isotope analyses can be pushed further to better understand individual life histories. By sampling different skeletal tissues of an individual, which formed at different periods of their life, it becomes possible to observe how their isotopic composition, and therefore diet and geographical mobility, varied throughout their lifetime [16]. Increasing the sample count can also increase the resolution of isotopic variation and give a better understanding of life histories.

Recreating dietary histories at a substantially higher resolution is possible, through the incremental sampling of tooth dentine for isotope analysis [14]. Incremental isotope analysis is based on the fact that human dentine forms in a sequential manner, from the tip of the dentine horn down to the root apex [30]. As the dentine is forming, it locks in the isotopic composition of the dietary intake at that age, effectively tracking the isotopic changes in the system throughout dentine formation [26]. This is particularly useful when identifying changing diets relating to age or season, and for determining the duration of exclusive breastfeeding and the weaning process [14, 15]. Multi-sampling techniques have previously been implemented in the region, specifically to reconstruct nursing practices [26, 28].

For the people of Legaire Sur, $\delta^{13}C$, $\delta^{15}N$, $\delta^{18}O$, $^{87}Sr/^{86}Sr$, $\delta^{88}Sr$, and [Sr] analysis coupled with incremental sampling techniques provides a unique opportunity to further reconstruct life histories during the Late Neolithic-Chalcolithic transition of north-central Iberia. With notable isotope research performed in nearby regions, comparative analysis of the Legaire Sur population with contemporaneous populations is possible, leading to an understanding of not only intra-population dynamics but also how this community aligns and connects with the wider north-central Iberian populations.

Legaire Sur & Legaire Megalithic Park

The Legaire Megalithic Park (42.838100, -2.270800; WGS84) was an important landscape in late prehistoric times. Situated in the Álava region of the Basque Country, north-central Iberia, the site holds evidence of continuous use from at least the Late Neolithic, through to the Iron Age, with evidence of re-use in later periods [31]. The landscape boasts a plethora of megalithic monuments, archaeological artefacts, and both human and faunal remains. Among the monuments are ∼100 earth mounds, 14 menhirs, a stone circle, one cist, one chambered tomb, and one passage tomb, Legaire Sur, which is the focus of this study.

The Legaire Sur passage tomb was first excavated in 1925 by E. Eguren [32]. This early work revealed that the monument, composed of clay and limestone slabs, comprised a main and a secondary chamber, with an associated short corridor leading from the southeast of the mound. A MNI (minimum number of individuals) of 9 were found in these excavations, though the remains have since been lost. Following this initial investigation, the monument was believed to be a total of 19m2. The dolmen was re-excavated in 2019 and 2020, as part of a project focusing on the re-investigation and preservation of Legaire monuments, promoted by the Museums and Archaeology Service of the Álava Provincial Council (DFA) and co-financed by the Gondra-Barandiarán Foundation [33, 34].These modern excavations offered a re-interpretation of the tomb: the 'main chamber' defined by Eguren is now considered part of the corridor, whereas the 'secondary chamber' is part of a larger main chamber of 4,5m^2 that had not been excavated and, therefore, remained intact. Archaeological evidence found within this chamber includes ceramics, lithics, bone tools and faunal remains, including a complete canid skeleton, as well as a human skeletal assemblage comprising ca. 8,000 bone elements.

These human bones were found mostly commingled (except for four partially connected skeletons found on the floor of the chamber) and very fragmented. Their study provided a minimum number of 25 individuals, mostly identified through mandibles or, in some cases particularly among juveniles, by other elements considering their different age-at-death estimation. All age groups, including one foetus, and both sexes are represented within the population [34].

Radiocarbon dating performed on 10 human bone samples from the chamber indicates that the site was used for burial between ca. 3400 and 2300 BC, between the Late Neolithic and the Late Chalcolithic periods (Table 1). Interestingly, the radiocarbon date obtained from the

Table 1. Published radiocarbon dates from Legaire Sur (10 humans, 1 domestic dog) [33, 34].

Inventory number	Element	Reference	^{14}C (BP)	±	Cal BC (95%) (OxCal)		$\delta^{13}C$ (‰) (VPDB)	$\delta^{15}N$ (‰) (AIR)
					From	To		
SE12-19.166	Human skull	BETA-538423	3930	30	2558	2300	-20.4	8.7
SE12-19.356	Human skull	BETA-538422	4460	30	3337	3021	-19.9	9.3
SE12-19.437	Canid jaw	BETA-538421	1020	30	968 (AD)	1147 (AD)	-17.9	7.6
SE12-19.510	Human skull	BETA-538419	4180	30	2887	2666	-21.2	8.9
SE12-19.513	Human skull	BETA-538420	4450	30	3336	2945	-19.7	9.5
SE12-20.115	Human skull	OxA-V-3099-9	4429	22	3321	2929	-20.5	9.1
SE12-20.269	Human skull	OxA-V-3099-10	4427	21	3318	2928	-20.4	8.9
SE12-20.271	Human skull	OxA-V-3099-11	4506	21	3347	3101	-20.3	9.2
SE12-20.383	Human skull	OxA-V-3099-12	4027	21	2619	2471	-20.8	9.4
SE12-20.1593	Human vertebra	OxA-V-3099-7	4506	22	3348	3101	-20.5	9.1
SE12-20.1607	Human vertebra	OxA-V-3099-8	4499	23	3344	3099	-20.4	9.5

https://doi.org/10.1371/journal.pone.0316387.t001

complete canid skeleton indicates a far later, historical date (10th-12th centuries AD), potentially indicating the re-use of the site for a later burial of the animal.

Materials and methods

In this study, 18 of the 25 individuals from Legaire Sur were selected for life history reconstruction through multi-isotope analysis, as sufficient skeletal material for analysis was available from these individuals. They were all recovered from the main, intact chamber during the 2019 and 2020 investigations. A bone sample was taken from each individual for collagen extraction for bone collagen carbon ($\delta^{13}C_{bcol}$) and nitrogen ($\delta^{15}N_{bcol}$) isotope analysis (bcol = bone collagen). The majority of samples were taken from mandibles, specifically a fraction of the alveolar bone, except for those individuals represented through other body elements; three humeri (SE12-19.492, SE12-20.1602, and SE12-20.1619) and a coxal bone (ischium) (SE12-20.664). In addition, eight first (M1), six-second (M2) and five-third (M3) permanent molars and two deciduous second molars (dM2) from nine of those individuals were selected for both bulk enamel and incremental dentine isotope analyses since they were the only chronologically non-overlapping dental remains. A full list of which teeth were sampled per individual is provided in Table 2. Bulk enamel was taken from every sampled tooth to measure carbon ($\delta^{13}C_{ap}$) and oxygen ($\delta^{18}O_c$) isotope ratios of the carbonate fraction, as well as for strontium isotope ratios ($^{87}Sr/^{86}Sr$ and $\delta^{88}Sr$) and strontium concentrations ([Sr]). Incremental dentine isotope analysis was performed on all fully formed M1, M2, and M3s, to track potential changes in collagen carbon ($\delta^{13}C_{dcol}$) and nitrogen ($\delta^{15}N_{dcol}$) isotope ratios across each tooth formation period (dcol = dentine collagen). Where available, multiple teeth were taken from each individual (max: 3) to best track isotope changes related to mobility and diet over an individual's lifetime, with dentine and enamel of each molar type forming at different age ranges in childhood/adolescence [35]. Table 2 displays every individual analysed in this study, which elements were selected for analysis, and their estimated age-at-death and biological sex. No permits were required for the described study, which complied with all relevant regulations. The remains are now held in the Bibat Museum of Vitoria-Gasteiz (Álava, Basque Country, Spain) with the very same ID numbers referred to in this paper.

Table 2. List of individuals analysed, with age-at-death and sex estimations (this study), and which skeletal elements and teeth were selected for isotope analyses. X = the enamel was bulk sampled and the dentine was incrementally sampled. **O** = only the enamel was available for isotope analysis.

Individual [1]	Age-at-Death (years)	Sex [2]	Bone Element	dM2	M1	M2	M3
SE12-19.405	45–55	F	Mandible		X	X	X
SE12-19.492	13–20	Indet.	Right humerus				
SE12-19.515	30–35	Indet.	Mandible		X	X	X
SE12-19.536	>45	Indet.	Mandible				
SE12-20.486	40–45	M	Mandible			X	X
SE12-20.487	35–45	M?	Mandible		X	O	X
SE12-20.574	Adult	Indet.	Mandible				
SE12-20.664	<1	Indet.	Left ischium				
SE12-20.758	∼ 3	Indet.	Mandible	O	O		
SE12-20.1163	∼7.5	Indet.	Mandible	O	O		
SE12-20.1181	9±3	Indet.	Mandible				
SE12-20.1267	∼ 20	Indet.	Mandible				
SE12-20.1288	35–40	F	Mandible		X		
SE12-20.1289	>50	M	Mandible				
SE12-20.1334	20–24	F?	Mandible		X	X	
SE12-20.1373	40–45	M	Mandible		X	X	X
SE12-20.1602	13–20	Indet.	Left humerus				
SE12-20.1619	13–20	Indet.	Right humerus				

[1] The codes read as follows: SE12 is the site, 19 or 20 is the excavation campaign year (2019 vs. 2020), and then comes the inventory number given to the bone element that represents an individual (after MNI calculation).

[2] Where: *M* = male; *M*? = probable male; *F* = female; *F*? = probable female; *Indet*. = indeterminate.

https://doi.org/10.1371/journal.pone.0316387.t002

Age-at-death and biological sex estimation

Where possible, non-adult age was estimated based on dental development [35]. The grade of ossification of post-cranial bones [36] and the size of long bones were used as secondary criteria [37–39]. For adult individuals, the recommendations of [40] were applied, where possible. In the specific case of mandibles, dental wear [37] was used for age estimation and, where there was an associated cranium, this was completed with the analysis of cranial suture obliteration [41].

With regard to sex, no attempt was made to estimate the sex of infants and children, due to the difficulties involved in estimating sex in skeletally immature individuals. For adults and juveniles older than 15 years of age, estimation mainly focused on the morphology of mandibles and, where available, associated [36, 40], in the absence of associated hip bones. In the case of mandibles, the dimorphic features of the gonial angle, chin shape, mandibular ramus flexure and mental eminence were recorded. In the case of crania, those of the glabella, forehead shape, postzygomatic arch, nuchal crest, orbital margins, supraorbital ridges and mastoid processes were recorded.

Bulk enamel sampling

A powdered enamel bulk sample was taken from each tooth for carbon, oxygen, and strontium isotope analyses, as well as strontium concentrations. Before extraction, to limit possible diagenetic contamination, the outer 200–300μm of enamel was removed [25] using a handheld drill with a diamond drill headpiece. Bulk samples were consistently sampled, using a TACKlife rotary drill, from the same location from each tooth: the disto-lingual cusp. Samples were taken from the top of the crown down the singular cusp to ∼0.5mm above the enamel-root

junction (ERJ), in a vertical line (1.5mm wide) down the corner of the cusp. It is important to note that this method was designed as it best averages the isotope signals from the beginning to the end of the tooth enamels formation (ca. 2 to 4 years; [42]). Sampling of the complete crown in this manner, following the molar enamel growth patterns, limits the seasonal/age-related biases in the isotope values. Specifically, this method minimises the impact of seasonality on $\delta^{18}O$ values [43, 44].

>10mg of powdered enamel was collected ready for pre-treatment before analyses through mass spectrometry to remove all potential contaminations. Pre-treatment followed [45, 46], with enamel samples being treated with 2mL 0.1M of unbuffered acetic acid for <30 minutes. Samples were then rinsed three times with MilliQ and dried overnight in an oven at 50˚C.

Carbonate oxygen and carbon isotope analysis

Each pre-treated enamel sample was measured in duplicate. 1mg of sample was placed into glass vials in a Nu Perspective Isotope Ratio Mass Spectrometer (IRMS) with a NuCarb carbonate preparation device, at the Archaeology, Environmental Changes & Geo-Chemistry Research Group (AMGC), Vrije Universiteit Brussel (VUB). Samples were measured is duplicate (2x1mg) alongside both in-house and international standards; lso-Analytical standard IA-R022 (calcium carbonate), international (IAEA-603, NBS18, IAEA-CO-8) and in-house bioapatite standard (calcined bone (CBA): see [47]). Repeated measurements of the IAEA-603 (calcite) standard yield an average of 2.54 ± 0.19‰ and -2.36 ± 0.19‰ (n = 11, 1SD) $\delta^{13}C$ and $\delta^{18}O$ respectively. The calcium carbonate standards NBS18 (n = 3; 1SD) yield values of -4.96 ± 0.18‰ and -23.20 ± 0.26‰ for $\delta^{13}C$ and $\delta^{18}O$ respectively. The IAEA-CO-8 (calcite) standards returned an average of -5.76 ± 0.12‰ and -22.75 ± 0.25‰ (n = 10, 1SD) for δ13C and δ18O. For the internal calcined bone standard CBA, the $\delta^{13}C$ and δ18O values obtained are -14.45 ±0.33‰ for $\delta^{13}C$ and -10.93 ±0.19‰ for $\delta^{18}O$.

Strontium isotope analysis

Approximately 7 mg of pre-treated enamel powder from each sample was weighed into individual Teflon beakers, for Sr isotope analysis. Digestion, and subsequent column chemistry, were performed by hand under a class A 100 laminar flow hood, in a class 1000 clean room at AMGC-VUB. Analytical blanks were also processed following the same method. Samples were digested in 2 mL sub-boiled 14M HNO_3 at 90˚C for ~24 hours. After digestion, samples were dissolved in 2.5 mL of 7M HNO_3, of which 0.5 mL was extracted for strontium concentration analysis. Following appropriate dilution, the concentrations of Ca and Sr were determined using an Agilent 8900 (Advanced Applications configuration) tandem inductively coupled plasma mass spectrometer (ICP-MS/MS) at AMGC-VUB. The mass spectrometer was operated in MS/MS mode and the collision/reaction cell was pressurised with O_2 to reduce spectral interferences and improve stability and precision (e.g., [48]). Nuclides ^{44}Ca, ^{86}Sr, ^{88}Sr, and ^{115}In were monitored on mass. Quantification was performed relying on external calibration versus mixed standard element solutions, after appropriate dilution with 0.42 M HNO_3 and the addition of indium (In) as an internal standard. The obtained strontium (Sr) concentration data were normalised to 40 wt% Ca, as this is the concentration of Ca in hydroxyapatite (see [23]). All [Sr] mentioned in the text and supplementary data refer to Sr concentrations in ppm normalised to 40 wt% Ca. To evaluate the accuracy of the applied procedure, certified reference materials NIST SRM1400 (bone ash powder), NIST SRM1486 (bone meal powder), and NIST SRM1515 (apple leaves powder) were analysed in parallel. Based on repeated measurements of both samples and reference materials, the analytical precision of the procedure is estimated to be better than 5% relative standard deviation (1RSD).

For isotope analyses, strontium was extracted from the digested samples through column chromatography following the procedure described in [9, 49] using ion exchange resin (Sr-Spec, Triskem). In brief, the columns and resin were rinsed 2 times with 2M HNO_3 and then conditioned with 2 x 1 mL 7M HNO_3. Samples were then loaded in 4 steps of 0.5 mL 7M HNO_3 and rinsed using 5 x 1 mL 7M HNO_3. Finally, Sr was collected with 6 x 1 mL 0.05M HNO_3, and subsequently evaporated to dryness. Strontium isotope measurements of the purified enamel samples were carried out at VUB using a Nu Plasma 3 MC-ICP-MS (PD017 from Nu Instruments, Wrexham, UK), equipped with 16 Faraday cups, and 10^{11} Ω resistors.

ln the $^{87}Sr/^{86}Sr$ data were corrected for mass fractionation by internal normalisation to an international convention value ($^{86}Sr/^{88}Sr$ = 0.1194, IUPAC data [50]). The raw data were normalised using a standard-sample bracketing method with the recommended value of $^{87}Sr/^{86}Sr$ = 0.710248 [51]. Procedural blanks were considered negligeable (total Sr (V) of max 0.02 versus 7–10 V for analyses, equivalent to roughly 0.2%). For each sample, the $^{87}Sr/^{86}Sr$ value is reported with a 2SE error (absolute error value of the individual sample analysis–internal error). Repeated measurements of the NIST SRM 987 standard solution and NIST SRM 1400 yielded a mean $^{87}Sr/^{86}Sr$ value of 0.710246 ±0.000026 (n = 31, 2SD) and 0.713118 ±0.000037 (n = 17, 2SD) respectively. This is consistent with the mean value for NIST SRM 987 of 0.710252 ±0.000013 (2SD for 88 analyses) obtained by Thermal Ionisation Mass Spectrometry (TIMS; [51]) and the value of 0.713120 ±0.000033 (2SD; n = 6; [52]).

The results for $\delta^{88}Sr$ were simultaneously obtained by Zr-doped sample-standard bracketing following the methods described in [9]. These methods include intensive data screening and mass bias correction. During this analysis, repeated measurements of NIST SRM 1400 returned an average value of -0.326 ± 0.062 (n = 17, 2SD), close to the long-term of -0.329 ±0.061‰ (n = 318, 2SD; [9]).

Carbon and nitrogen isotope analyses

A full overview of the bone collagen extraction protocol is given in the Supporting Information: SECTION 1 in S1 File. Incremental dentine sampling followed the [16] 'puncturing' methodology, detailed in the publication, and also in [15]. In this study, a 2mm-wide mid-tooth longitudinal slice was taken from the M1s, M2s, and M3s selected for incremental dentine study (see Table 2), using an IsoMet1000® precision saw. Once this slice was demineralized, a 1.2mm diameter Harris Uni-core biopsy punch was used to take the incremental samples from cusp to apex from either the mesial or distal side of the tooth slice and down the centre of the primary dentine growth axis, being careful to not include any secondary or tertiary dentine. Taken dentine samples were given a putative age estimation following [16] after [35]; which estimates primary dentine formation rates, and, as such, age ranges, based upon the growth striations of dentine. This calculation is specific not only to the molar type (M1, M2, or M3), but also to the location of the sample on the dentine growth axis (crown, neck, superior half of the root, and inferior half of the root). Utilising these equations, the 1.2mm biopsy punch used represents a putative average of ∼ 300 days of growth across all teeth [53, 54].

Both dentine and bone collagen samples were measured on a Nu Horizon 2 Isotope Ratio Mass Spectrometer (Nu Instruments, Wrexham, UK) combined with a Eurovector Elemental Analyzer. All bone collagen samples were measured in duplicates and dentine samples in singular. Sample positions were randomised, with the samples being measured along with the Iso-Analytical standard IA-R069 (tuna protein) (n = 106, average and 1SD of -14.33±0.34‰ and +6.38±0.22‰ for $\delta^{13}C$ and $\delta^{15}N$ respectively), and international calibration standards IA-R068 (soy protein) (n = 15, average and 1SD of -20.52±0.56‰ and -4.42±0.60‰ for $\delta^{13}C$ and $\delta^{15}N$ respectively), IAEA-CH-6 (sucrose) (n = 16, average and 1SD of -6.24±0.71‰ for

$\delta^{13}C$), USGS88 (marine collagen) (*n* = 18, average and 1SD of -4.42±0.60‰ and -20.52 ±0.56‰ for $\delta^{13}C$ and $\delta^{15}N$ respectively), and USGS89 (porcine collagen) (*n* = 18, average and 1SD of -11.72±0.62‰ and 9.31±0.66‰ for $\delta^{13}C$ and $\delta^{15}N$, respectively). The reproducibility standard SRM3 (gelatin) (*n* = 14; 1SD) yield an average of reproducibility of -15.14±0.25‰ and 4.92±0.15‰ for $\delta^{13}C$ and $\delta^{15}N$ respectively. Which correspond well with the expected values of -15.3‰ and 5.12‰ for $\delta^{13}C$ and $\delta^{15}N$ respectively. For gluten standard (SRM4) (*n* = 11), the average and 1SD reproducibility is -26.62±0.23‰ and 5.20±0.07‰ for $\delta^{13}C$ and $\delta^{15}N$. Which correspond well with the expected values of -26.77‰ and 5.24‰ for $\delta^{13}C$ and $\delta^{15}N$ respectively.

Following [55], the ratio of C:N was calculated and analysed to reveal the extent of post-depositional alteration to collagen $\delta^{15}N$ and $\delta^{13}C$ values. DeNiro states that samples with a C:N range of 2.9–3.6 are typical of *in-vivo* collagen, with ratios falling outside this range indicating contamination. All bone collagen samples presented ratios within this range. Bone collagen values are presented alongside their %C and %N collagen yields, to ensure measured values were not affected by poor preservation (all samples were between 15.3%C to 47%C and 5.5%N to 17.3%N, indicating satisfactory, repeatable values [56]). 11 incremental dentine samples did not meet this quality control, and were subsequently removed from the dataset. $\delta^{13}C$ and $\delta^{15}N$ values are given in Table 4 (average and range of dentine increments) and Table 5 (bulk bone collagen values).

Results

Bulk enamel results

The $^{87}Sr/^{86}Sr$, $\delta^{88}Sr$, $\delta^{13}C_{ap,}$ $\delta^{18}O_c$ and [Sr] values for all 21 teeth analysed are listed in Table 3, with the variable correlations being graphically represented in Figs 1 and 2. The average $^{87}Sr/^{86}Sr$ value found in the tooth enamel from Legaire Sur is 0.7082 ±0.0012 (2SD). Interestingly, when comparing $^{87}Sr/^{86}Sr$ value variation intra-individually, there is minimal variation between teeth (average individual variation: 0.0002). Inter-individual sees a far greater range of values (0.0020, from 0.7077 to 0.7096). However, this range is largely influenced by the comparatively higher values of individual SE12-19.405, a biological female (M1: 0.7095, M2: 0.7096, M3: 0.7092, Fig 1). After removing outlier SE12-19.405, values reveal a smaller range of values (0.0011, from 0.7077 to 0.7088). A similar trend is observed in the $\delta^{88}Sr$ values, with SE12-19.405 having the most depleted values (M1: -0.594‰, M2: -0.716‰, M3: -0.616‰, though not statistically an outlier Fig 1). The average $\delta^{88}Sr$ for the site is -0.465‰, with a range of -0.716‰ to -0.217‰. This range significantly decreases to -0.581‰ to -0.217‰, if SE12-19.405 is removed from the population. The intra-individual range is comparatively low within $\delta^{88}Sr$ profiles, with an average variation between teeth being 0.147‰ $\delta^{88}Sr$.

$\delta^{13}C_{ap}$ and $\delta^{18}O_c$ profiles also present a similar trend to both the $^{87}Sr/^{86}Sr$ and $\delta^{88}Sr$ profiles, with individual SE12-19.405 having values notably different from the rest of the population. The average $\delta^{13}C_{ap}$ is -14.0 ±0.8‰, with a site range of -16.3‰ to -12.9‰ (IQR1.5) (reduced to -14.5‰ to -12.9‰, if SE12-19.405, a statistical outlier, is removed). Intra-individual $\delta^{13}C_{ap}$ variation is always $<$1.0‰, except for SE12-20.1163 which has a range of 1.6‰, which is still considered a non-significant variation [43].

Individual SE12-19.405 presents notable intra-individual $\delta^{18}O_c$ value variation; M1: -4.0‰, M2–3.8‰, M3: -5.9‰. Whilst SE12-19.405's $\delta^{18}O_c$ values do cluster close with the site range of values; -6.3‰ to -3.8‰ (average: -5.2 ±0.8‰), their M1 and M2 do present the highest values in the population (Fig 2). Comparing the $\delta^{18}O_c$ values to the other apatite isotope proxies reveals no significant correlations (Fig 2).

Table 3. $\delta^{13}C_{ap}$, $\delta^{18}O_c$, $^{87}Sr/^{86}Sr$, $\delta^{88}Sr$, and [Sr] values from human tooth enamel.

Tooth	Age-at-Death (years)	Sex [1]	$^{87}Sr/^{86}Sr$	2SE	$\delta^{88}Sr$ (‰)	[Sr] [2,3] (ppm)	$\delta^{13}C_{ap}$ [4] (VPDB) (‰)	$\delta^{18}O_c$ [4] (VPDB) (‰)
SE12-20.1288.M1	35–40	F	0.708332	0.000009	−0.456	60	−14.15	−5.65
SE12-20.1334.M1	20–24	F?	0.707788	0.000010	−0.344	183	−13.33	−5.71
SE12-20.1334.M2			0.707582	0.000011	−0.530	186	−13.03	−6.05
SE12-20.1373.M1	40–45	M	0.707905	0.000011	−0.544	112	−14.31	−4.62
SE12-20.1373.M2			0.707739	0.000009	−0.421	172	−13.77	−4.92
SE12-20.1373.M3			0.707692	0.000010	−0.283	195	−13.68	−5.47
SE12-19.405.M1	45–55	F	0.709552	0.000011	−0.594	84	−15.79	−4.02
SE12-19.405.M2			0.709631	0.000009	−0.716	97	−16.32	−3.80
SE12-19.405.M3			0.709251	0.000008	−0.616	120	−14.29	−5.78
SE12-20.486.M2	40–45	M	0.707668	0.000010	−0.452	184	−14.13	−6.22
SE12-20.486.M3			0.707908	0.000007	−0.426	175	−14.43	−6.34
SE12-20.487.M1	35–45	M?	0.708058	0.000010	−0.217	108	−13.87	−4.24
SE12-20.487.M2			0.707771	0.000009	−0.380	114	x	X
SE12-20.487.M3			0.707996	0.000008	−0.542	86	−13.95	−4.91
SE12-19.515.M1	30–35	Indet.	0.708414	0.000009	−0.389	69	−14.03	−5.62
SE12-19.515.M2			0.707908	0.000012	−0.422	41	−13.57	−5.98
SE12-19.515.M3			0.708503	0.000010	−0.448	136	−13.57	−6.00
SE12-20.1163.dM2	∼7.5	Indet.	0.708132	0.000007	−0.380	87	−12.86	−4.42
SE12-20.1163.M1			0.708074	0.000010	−0.511	x	−14.46	−5.13
SE12-20.758.dM2	∼ 3	Indet.	0.708784	0.000007	−0.581	98	−13.49	−4.66
SE12-20.758.M1			0.708248	0.000006	−0.512	139	−13.32	−4.45

[1] Where: *M* = male; *M*? = probable male; *F* = female; *F*? = probable female; *Indet*. = indeterminate.

[2] SE12-20.1163's M1 did not yield a valid [Sr] value.

[3] [Sr] data is normalised to 40wt.% [Ca]. [23]

[4] X = values that were unobtainable due to limited sample quantity

https://doi.org/10.1371/journal.pone.0316387.t003

The [Sr] concentration values present a range of values from 41ppm to 195ppm, with an average of 122ppm. Individuals SE12-20.1334 (average: 185ppm), SE12-20.486 (average: 180ppm), and SE12-20.1373 (average: 159ppm) present the highest [Sr] in the population, compared to individuals SE12-20.1288 (M1: 60ppm) and SE12-19.515 (M1: 69ppm, M2: 41ppm). Intra-individual [Sr] variation is mixed across individuals, with the average variation being 42ppm. Individual SE12-19.515 presents the largest intra-individual variation (95ppm), with a substantial increase in values from M1/M2 to M3. The lowest intra-individual variations are observed in two individuals with some of the lowest [Sr] values, SE12-20.1334 (variation: 3ppm) and SE12-20.486 (variation: 9ppm).

Incremental dentine collagen results

An overview of the average and range of $\delta^{13}C_{dcol}$ and $\delta^{15}N_{dcol}$ values for all incrementally sampled individuals, according to tooth type, is available in Table 4. A total of 7 out of 217 dentine increments extracted from the molars failed to meet the collagen quality criteria, leaving 210 valid values. These values are presented in Fig 3, according to the individual, tooth type, and calculated increment age (following: [16]).

Across all individuals, a $\delta^{13}C_{dcol}$ values range from −21.7‰ to −19.2‰ is observed, with a total average value of $\delta^{13}C_{col}$ = −20.4 ±0.5‰. One individual (SE12-19.405) presents a comparatively lower $\delta^{13}C_{dcol}$ childhood sequence (total average = −21.0 ±0.4‰) than the others.

Table 4. The average and range of $\delta^{13}C$ and $\delta^{15}N$ values from the incremental sampling of human tooth dentine collagen.

Individual	M1: Range		M2: Range		M3: Range		M1: Average		M2: Average		M3: Average		Total Dentine Range		Total Dentine Average	
	$\delta^{13}C$	$\delta^{15}N$	$\delta^{13}C$	$\delta^{15}N$	$\delta^{13}C$	$\delta^{15}N$	$\delta^{13}C$	$\delta^{15}N$	$\delta^{13}C$	$\delta^{15}N$	$\delta^{13}C$	$\delta^{15}N$	$\delta^{13}C$	$\delta^{15}N$	$\delta^{13}C$	$\delta^{15}N$
SE12-20.1288	1.3	2.0					−20.5	+10.0					1.2	2.0	−20.5	+10.0
SE12-20.1334	0.4	2.5	0.7	1.4			−20.3	+8.6	−20.4	+8.4			0.7	2.5	−20.4	+8.5
SE12-20.1373	1.0	2.0	0.5	2.0	0.8	0.8	−20.4	+8.8	−20.3	+8.5	−20.4	+8.3	1.0	2.2	−20.4	+8.6
SE12-19.405	0.6	1.7	1.4	1.6	0.9	0.6	−21.2	+9.6	−20.9	+9.7	−20.9	+10.0	1.4	2.1	−21.0	+9.8
SE12-20.486			0.9	1.0	0.5	1.0			−20.3	+9.0	−20.3	+8.7	0.9	1.3	−20.3	+8.8
SE12-20.487	2.1	2.5			1.1	1.3	−19.9	+9.0			−19.8	+9.2	2.1	2.5	−19.8	+9.1
SE12-19.515	1.0	3.2	1.1	1.4	1.2	1.8	−20,0	+8.8	−20.0	+8.6	−19.9	+9.0	1.2	3.2	−20.0	+8.8
ALL	2.3	3.3	1.2	2.0	2.4	3.3	−20.4	+9.2	−20.2	+8.9	−20.2	+9.1	2.5	3.6	−20.4	+9.0

https://doi.org/10.1371/journal.pone.0316387.t004

$\delta^{15}N_{dcol}$ values present far greater variation, from +7.9‰ to +11.1‰ (range = 3.3‰), with an overall average of 9.1 ±0.7‰. Most individuals show a great deal of intra-individual $\delta^{15}N$ variation (average = 2.3‰, all of which >2.0‰). For individuals with an M1 (all excluding SE12-20.486), most of this intra-individual $\delta^{15}N_{dcol}$ variation is observed within the crown of the M1 (average $\delta^{15}N$ M1 variation = 2.3‰).

With regard to the profiles, it is noteworthy the existence of some discrepancies between the profiles of the different teeth in some individuals. In some cases, this is probably due to the chronological misalignment of the profiles (e.g., individual SE-12.20.1334), which may reflect differences between the actual timing of formation of the teeth and that theoretically expected [35]. In others, however, the important differences observed cannot be attributed to misalignment (e.g., SE-12.19.405, SE-12.19.515 and SE-12.20.1373). Nor they can be attributed to mass spec performance, since samples have been randomly measured. These discrepancies are age-

Table 5. $\delta^{13}C$ and $\delta^{15}N$ values from human bone collagen.

Individual	Bone Element	Age-at-Death (years)	Sex [1]	%N	$\delta^{15}N$ (AIR) (‰)	%C	$\delta^{13}C$ (VPDB) (‰)	C:N
SE12-19.405	Mandible	45–55	F	12.3	+9.21	34.2	−20.65	3.2
SE12-19.492	Humerus	13–20	Indet.	14.8	+8.52	40.4	−19.68	3.2
SE12-19.515	Mandible	30–35	Indet.	13.9	+8.61	38.1	−19.73	3.3
SE12-19.536	Mandible	>45	Indet.	14.2	+8.34	39.8	−19.99	3.2
SE12-20.486	Mandible	40–45	M	10.2	+7.89	27.6	−20.06	3.2
SE12-20.487	Mandible	35–45	M?	13.8	+8.92	37.5	−19.91	3.2
SE12-20.574	Mandible	Adult	Indet.	13.4	+9.00	36.8	−19.98	3.2
SE12-20.664	Coxal	<1	Indet.	14.5	+10.62	39.9	−20.28	3.2
SE12-20.758	Mandible	∼ 3	Indet.	13.8	+10.52	37.4	−19.74	3.2
SE12-20.1163	Mandible	∼7.5	Indet.	14.0	+8.66	38.7	−20.05	3.2
SE12-20.1181	Mandible	9±3	Indet.	14.7	+8.08	40.2	−20.51	3.2
SE12-20.1267	Mandible	∼ 20	Indet.	11.8	+7.78	32.1	−20.42	3.2
SE12-20.1288	Mandible	35–40	F	11.7	+8.86	32.0	−20.46	3.3
SE12-20.1289	Mandible	>50	M	14.1	+8.84	39.6	−19.97	3.3
SE12-20.1334	Mandible	20–24	F?	14.4	+8.30	40.1	−20.02	3.2
SE12-20.1373	Mandible	40–45	M	14.5	+8.66	40.3	−20.33	3.4
SE12-20.1602	Humerus	13–20	Indet.	14.3	+8.39	40.3	−19.81	3.2
SE12-20.1619	Humerus	13–20	Indet.	14.7	+8.65	40.2	−19.95	3.3

[1] Where: *M* = male; *M*? = probable male; *F* = female; *F*? = probable female; *Indet*. = indeterminate.

https://doi.org/10.1371/journal.pone.0316387.t005

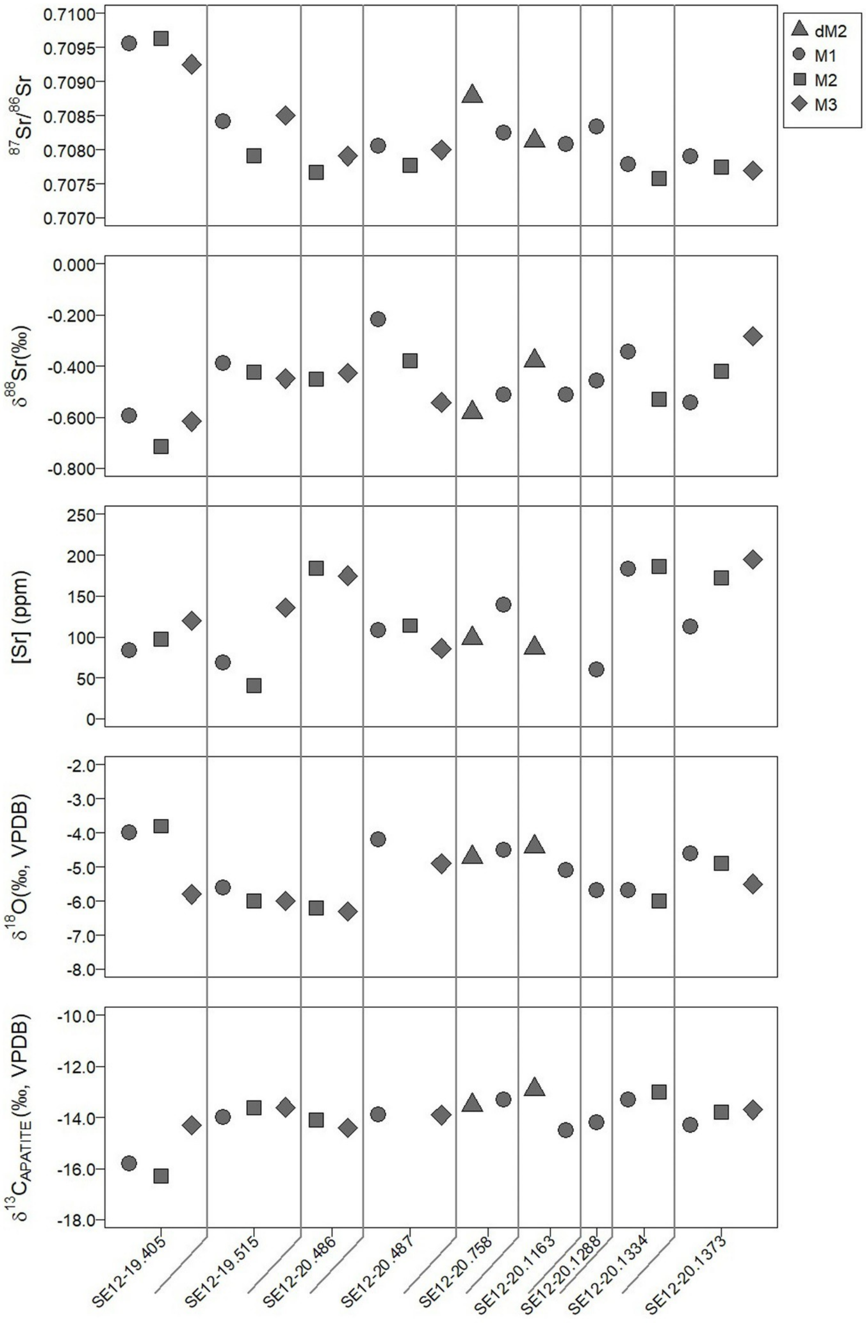

Fig 1. Comparison of $\delta^{13}C_{ap}$, $\delta^{18}O_c$, $^{87}Sr/^{86}Sr$, $\delta^{88}Sr$, and [Sr] values from human tooth enamel. Created in RStudio (version 2023.09.1 "Desert Sunflower"), using "plot()" function in the "base" package (version 4.0.5).

https://doi.org/10.1371/journal.pone.0316387.g001

section-specific and, considering the large and occasionally drastic shifts observed in isotope values, especially in carbon, are likely the result of capturing slightly different timings when micro-sampling teeth with very sinusoidal isotopic profiles.

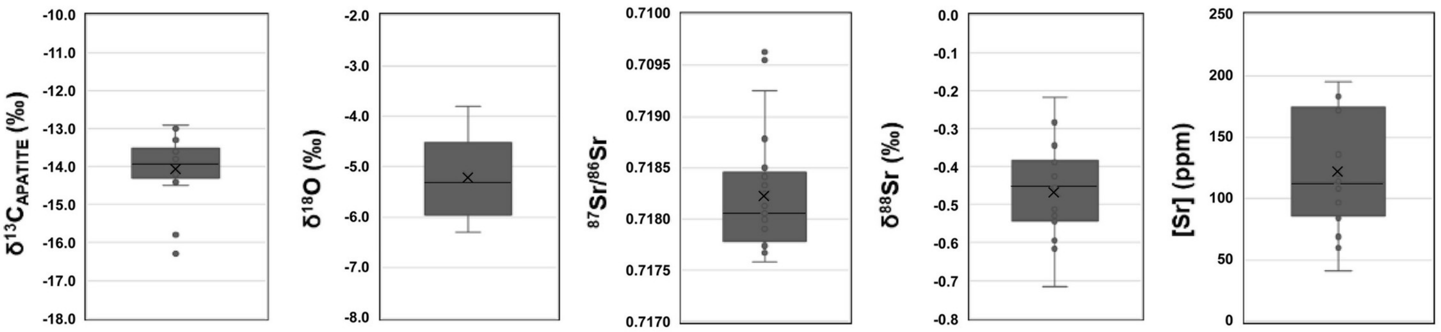

Fig 2. Box plots of $\delta^{13}C_{ap}$, $\delta^{18}O_c$, $^{87}Sr/^{86}Sr$, $\delta^{88}Sr$, and [Sr] values from human dental enamel. In each plot, the X = average value, line = median value.

https://doi.org/10.1371/journal.pone.0316387.g002

Bulk bone collagen results

In total, 18 $\delta^{13}C_{bcol}$ and $\delta^{15}N_{bcol}$ values were obtained, presented in Table 5 and Fig 4. The $\delta^{13}C_{bcol}$ values all cluster relatively close together, ranging from -20.9‰ to -19.7‰, range = 1.2‰, with an average $\delta^{13}C_{bcol}$ value of -20.1 ±0.3‰. A greater range is observed in the $\delta^{15}N_{bcol}$ values, from 7.9‰ to 10.6‰, range = 2.7‰. The average $\delta^{15}N$ is 8.8 ±0.7‰, with most individuals clustering between 8.0‰ – 9.0‰. However, two individuals younger than 3 years of age present values >1.5‰ higher than this range (SE12-20.664: 10.6‰, SE12-20.758: 10.5‰). There does not appear to be any grouping between the bone collagen samples taken from mandibular or humeri samples; however, the most ^{15}N enriched individual (SE12-20.664) is represented by the only pelvic bone sample in the analysis, which corresponds to a baby younger than 1 year of age.

A bioavailable $^{87}Sr/^{86}Sr$ baseline (BASr) for north-central Iberia

The bioavailable strontium baseline is an expansion of the previously defined classes by [27]. Using the Geología GEODE 50k [57] as a base, the prediction per geological unit was expanded. The datapoints used to create this BASr are located >30km south and west of the site, with 87Sr/86Sr values ranging from 0.7078–0.7104. Unfortunately, there is no available local BASr at the site and to the north and east, thus limiting interpretations of the human $^{87}Sr/^{86}Sr$ data. Whilst the site shares similar bedrock (predominantly a mixture of Miocene and Oligocene sandstone, Mesozoic limestone, conglomerates, and sand) with the regions sampled for baseline values (Fig 5), it should be noted that there are different bedrock lithologies and ages to the north and east which may have different bioavailable $^{87}Sr/^{86}Sr$.

Determining a $\delta^{13}C_{bcol}$ and $\delta^{15}N_{bcol}$ baseline

One setback for the interpretation of this site is the scarcity of faunal remains. As such, a site-specific dietary $\delta^{13}C$ and $\delta^{15}N$ baseline cannot be determined. Legaire Megalithic Park does not hold evidence for coeval prehistoric settlements, and therefore there is a lack of consumption waste. A dietary baseline was constructed instead using neighbouring (Basque Country region, ~50km), contemporaneous faunal baselines, taken from previous publications. This includes faunal values from the Mesolithic/Neolithic site of Santimamiñe (n = 8) (~65km away) and the Chalcolithic site of Pico Ramos (n = 4) (~88km away), [29]. A further 5 Late Neolithic/Chalcolithic cave sites; Peña Larga (n = 4), El Sotillo (n = 1), Los Husos I (n = 18), Alto de la Huesera (n = 2), Chabola de la Hechicera (n = 4), which are all located in Rioja Alavesa, Álava, (~35km from Legaire Sur), were used from [4]. A summary of this faunal data

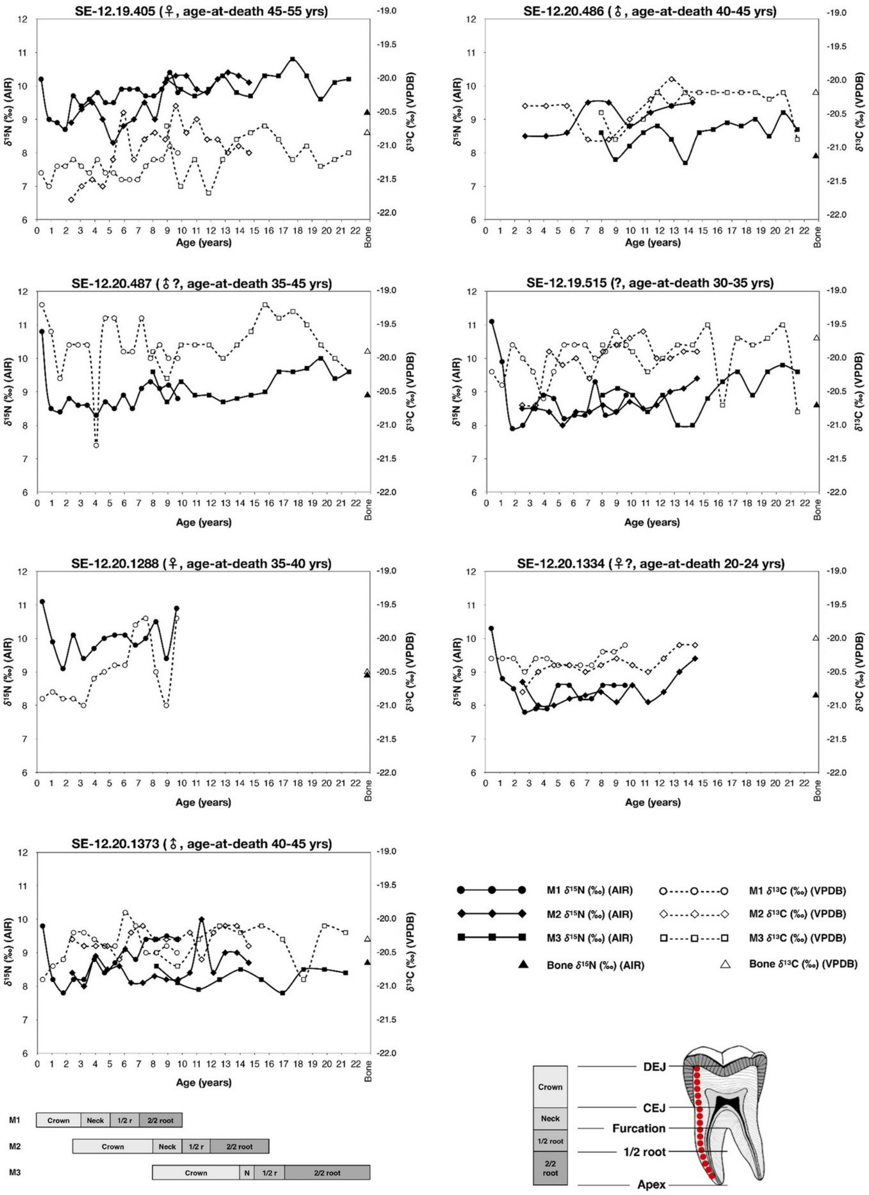

Fig 3. Incremental $\delta^{13}C$ and $\delta^{15}N$ values from dentine collagen plotted against estimated ages of tooth formation (following [16]).

https://doi.org/10.1371/journal.pone.0316387.g003

(*n* = 41) is given in the Supporting Information (S1 Table in S1 File), with the values and geographical coordinates.

For this generated faunal isotope baseline (Fig 5), an average $\delta^{13}C$ value of -20.7 ±0.5‰ and an average $\delta^{15}N$ value of 4.7 ±1.5‰ is seen. These values are consistent with the herbivorous consumption of plants in a temperate C_3 ecosystem [59]. The $\delta^{15}N$ values display a far greater range, from 2.6‰ to 8.7‰; range 6.1‰. The majority of the values cluster between 2.0‰-7.0‰, which is to be expected from herbivorous grazers [60]. However, there are some outliers. From [4], there are several individuals with values >6.0‰, including omnivorous domestic pigs (*Sus domesticus*), two red deer (*Cervus elaphus*), and a sheep/goat (*Ovis aries/Capra hircus*) whose high values were attributed to a potential nursing signal. Although this baseline

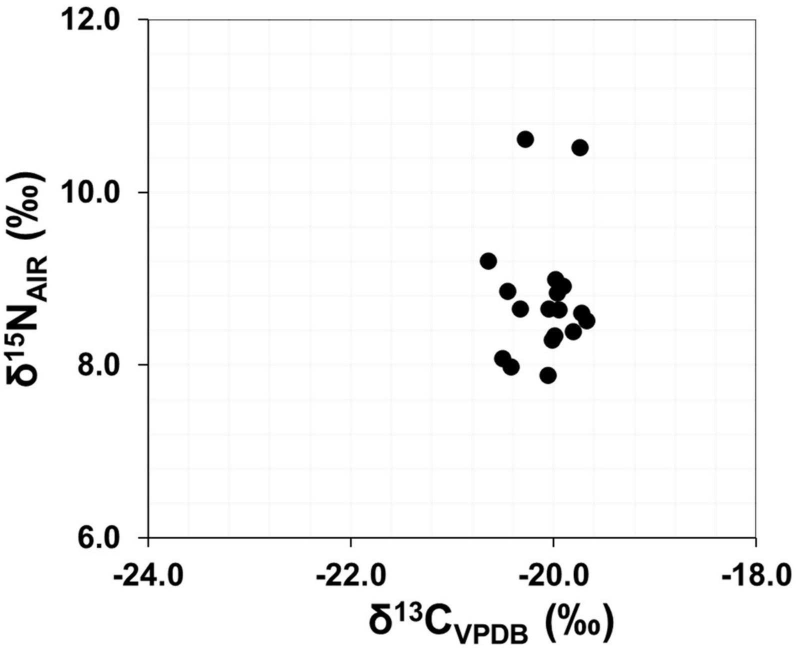

Fig 4. $\delta^{13}C$ and $\delta^{15}N$ values from the human bone collagen.

https://doi.org/10.1371/journal.pone.0316387.g004

includes a substantial range of $\delta^{15}N$, it is important to include potential nursing individuals within the study, as this diet may be reflective of what the people from Legaire Sur were also consuming.

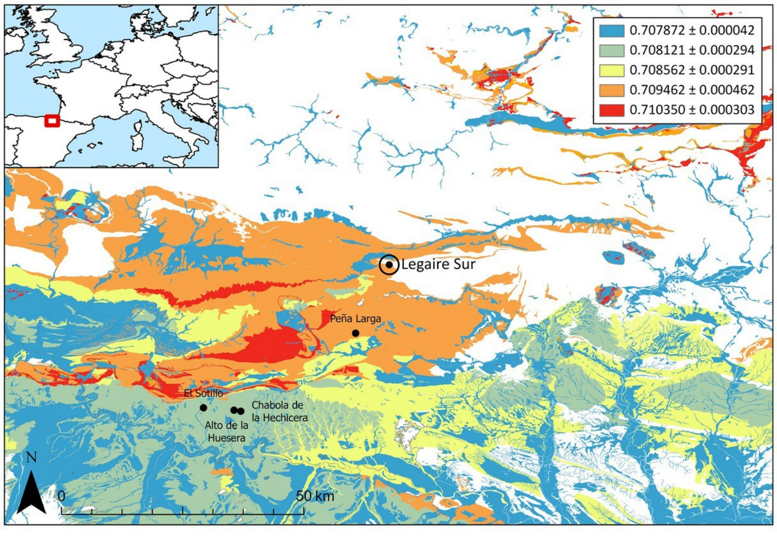

Fig 5. Regional 87Sr/86Sr bioavailable baseline for Legaire Sur based on modern plant data from [27]. The mean $^{87}Sr/^{86}Sr$ of each geological unit (based on Geología GEODE 50k [57]), is represented. $^{87}Sr/^{86}Sr$ values are given with composite SD. Legaire Sur is located with a double circle, alongside major contemporaneous sites from the Rioja Alavesa Valley (El Sotillo, Alto de la Huesera, Chabola de la Hechicera, and Peña Larga). The location of Legaire Sur and the baseline is highlighted on a modern geographical map [58].

https://doi.org/10.1371/journal.pone.0316387.g005

Discussion

Mobility histories

There is minimal $^{87}Sr/^{86}Sr$ intra-individual variation, which implies that these individuals all presented limited long-distance residency mobility to 13 years of age (the age when the formation of the crown of the third permanent molars finishes) [42]. Inter-individually, all individuals show close clustering for $\delta^{13}C_{ap}$, $\delta^{18}O$, $^{87}Sr/^{86}Sr$, and $\delta^{88}Sr$ values, aside from SE12-19.405. It is possible that SE12-19.405's childhood was likely spent away from the rest of the population. This is evidenced not only by the elevated $\delta^{18}O$ and $^{87}Sr/^{86}Sr$ values, but by the comparatively depleted $\delta^{13}C_{ap}$, $\delta^{88}Sr$, and $\delta^{13}C_{dcol}$ values. Whilst the $\delta^{13}C$ values still imply the consumption of C_3 plants, the comparative depletion, alongside the ^{88}Sr-depletion, suggests that those plants may have been cultivated in different landscapes or with different methods, or that animals may have been foddered with different plants. As such, whilst most individuals could be potentially 'locals', and spent their childhoods at a relatively close distance to the site, SE12-19.405 presents an 'outlier' life history, indicating she initially belonged to another group and may have joined the community in later life. Despite being only a singular case, SE12-19.405 being identified as a biological woman might be consistent with patrilocality documented at that time, not only in Rioja Alavesa (see [27]) but across Europe [61].

When compared to the previous work of Fernández-Crespo *et al.* [27] (Fig 6), a very similar population $^{87}Sr/^{86}Sr$ isotope average and range is observed between the individuals from Legaire Sur and individuals found within other megalithic graves in the Rioja Alavesa (∼30km south-west of the site), whereas they present a more constrained range of $^{87}Sr/^{86}Sr$ values than those uncovered from the funerary caves of that region, indicating separate communities raised in geologically distinct locations (aside from SE12-19.405). Further, much like in the Rioja Alavesa's megalithic graves, the only individuals with 'outlier' $^{87}Sr/^{86}Sr$ values were biological females, with identified males holding the most limited $^{87}Sr/^{86}Sr$ variation. While identifying biological sex in the Legaire Sur population is restricted, it is interesting to observe a similarity in trend.

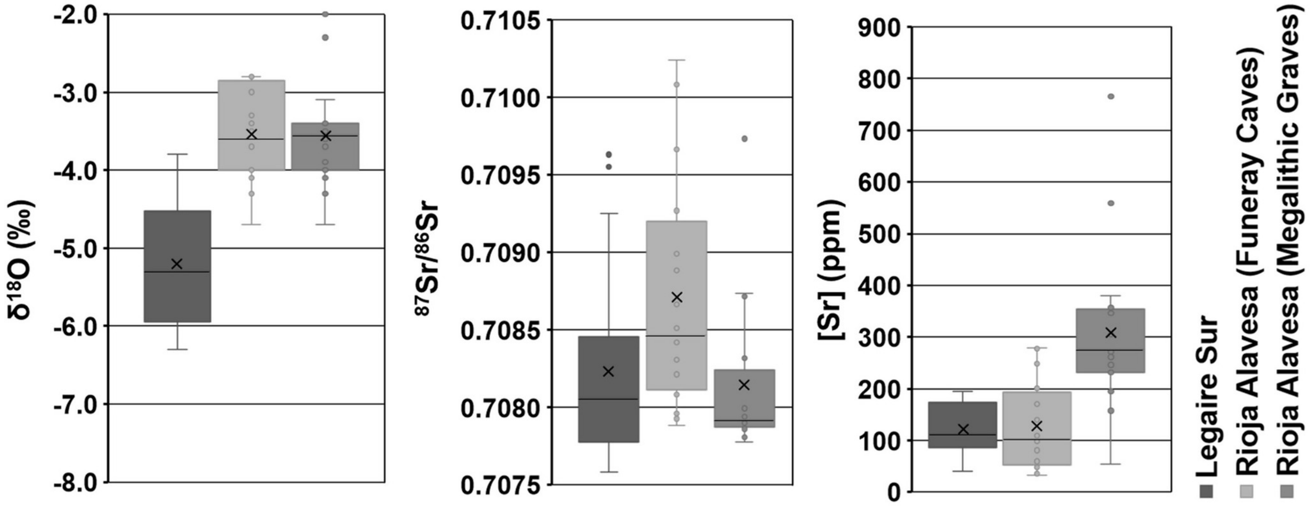

Fig 6. A comparison of the [Sr], $\delta^{18}O$, and $^{87}Sr/^{86}Sr$ data from all the human enamel apatite samples analysed from Legaire Sur with contemporaneous individuals analysed from funerary caves and other megalithic graves in [27]. (Legaire Sur $n = 21$, Funerary Caves $n = 16$ ($\delta^{18}O$: 32), Megalithic Graves $n = 16$ ($\delta^{18}O$: 32)). In each plot, the X = average value, line = median value.

https://doi.org/10.1371/journal.pone.0316387.g006

When comparing the $\delta^{18}O$ and [Sr] values of Legaire Sur and those of Rioja Alavesa's megalithic graves, notable differences are observed, conversely. The individuals from the megalithic graves analysed by Fernández-Crespo *et al.* [27] present comparatively higher $\delta^{18}O$ and [Sr] values than those from Legaire Sur.

The higher [Sr] values in Rioja Alavesa could be attributed to a lower proportion of meat consumption but the [Sr] are significantly higher and other reasons need to be considered. Firstly, the consumption of plants and food resources from a Sr-rich environment such as plants obtained close to a salt lake. Secondly, the significant consumption of salt, which can lead to the significant enrichment of the body's Sr-concentration [23] and which, in the case of Rioja Alavesa, might be supported by the presence at that time of ca. 50 salt lakes that could have been used for cultivation, herding, or other subsistence activities (see [27] for concentration data on plants and salt from the Alavsea region). Whilst, as said, the 87Sr/86Sr values from Legaire Sur do not differ significantly from those in the Rioja Alavesa Region suggesting perhaps a geologically similar region, their comparatively lower [Sr] indicate that this population did not exploit either area around salt lakes of the Ebro valley.

Comparatively lower $\delta^{18}O_c$ values in individuals from Legaire Sur may also indicate water consumption from different environments or sources. Legaire Sur is located $\sim$1150m above sea level, whereas the Rioja Alavesa valleys are located between 500-600m above. Previous research has indicated that increased altitude leads to a depletion in ^{18}O in precipitation [62]. Alternatively, this $\delta^{18}O_C$ difference may be down to the consumption of water from different sources (e.g., upland rivers vs. valley lakes) [62]. Either way, the results support the idea that those at Legaire Sur were consuming, and likely inhabiting, in a different type of environment to those from the Rioja Alavesa.

Mobility may also be suggested from incremental dentine isotope profiles, with the advantage that these provide finer temporal resolutions than bulk enamel studies. In the isotope profiles analysed in Legaire Sur, it is worth noting the unusually large, recurrent and occasionally drastic shifts observed in the carbon isotope values of some individuals (e.g., SE-12.19.405, SE-12.20.487, SE-12.19.515 and SE-12.20.1288), in some cases higher than 1.5‰. This sinusoidal pattern (see Fig 3), which has not been identified in Late Neolithic individuals from Rioja Alavesa [27], may reflect the seasonal exploitation of different landscapes, particularly if they feature different vegetation cover and altitude, as is the case of Legaire uplands vs. nearby Arakil valley lowlands (3km north of Legaire). It has been documented in the historical periods that local herders and their families used to live in the latter during the cold months and temporally moved up with their herds to Legaire from mid-spring to early autumn, behaviour that is replicated in other mountainous areas of Navarre and the Basque Country [63]. With this evidence in mind, it is tempting to speculate that late prehistoric people from Legaire Sur did the same, especially considering that life above 1,000m above sea level during the season of critical resource availability would have been hard. However, it is acknowledged that this conclusion, drawn from the dentine profiles alone, would be conjectural. Instead, these discussions are currently focused on the seasonal changes in diet and food availability, rather than the geographical location of said resources.

Dietary histories

Reconstructing nursing strategies through dentine collagen and enamel apatite. During an individual's early life, it is expected that they would be consuming breast milk, which would lead to higher $\delta^{15}N$ and $\delta^{13}C$ values. Consequently, it can be determined how long an individual was breastfed, and how soon complementary foods were introduced (weaning), with an expected progressive ^{15}N- and ^{13}C-depletion of around 2–3‰ and 0.5‰, respectively, at the

cessation of nursing [64, 65]. This information may be recorded in the first permanent molar (M1), with the dentine beginning to form around birth and completing formation between 9 to 10 years of age [35].

Our results do not allow the identification of isotope values clearly compatible with an exclusive breastfeeding signal in the first sample/s of the incremental profiles. This is not due to dental wear, since, despite the enamel crown of each M1 being worn, the dentine horn for all individuals remained largely untouched, maintaining the entirety of the dentine growth sequence. Since each 1.2mm puncture used in the sampling represents ca. 300 days of growth–the average rate of dentine growth (following the growth striae) in the M1 is ∼4μm per day [53, 54], the 1st increment represents the average isotope signal of barely the first year of life, merging both exclusive breastfeeding and weaning signals. This results in low ^{13}C- (average = 0.3‰, apart from SE12-20.487: 1.1‰) and ^{15}N-variations (average = 2.3‰) between the 1st and 2nd/3rd crown increments (Fig 3). This sustained decrease in δ^{13}C and δ^{15}N is observed until ca. on average 1.9 ±0.4 years, which suggests that the cessation of nursing was relatively early, since in most traditional societies weaning ends around 2.5 to 3 years of age [66]. Supporting this is the absence of ^{15}N-depletion observed in the M2s (which begin their formation at ∼2.5 years), except for individual SE-12.20.1334, indicating that weaning must have been completed before this age [35].

Evidence for weaning ages has previously been determined using δ^{13}C and δ^{18}O variation between teeth [67, 68]. Previous research has demonstrated that ingestion of breast milk should lead to a ∼0.5‰ decrease in δ^{13}C and a ∼0.5‰ increase in δ^{18}O values [69]. Therefore, if breastfeeding occurred during the formation of the M1, but the individual was fully weaned during/by the forming of the M2, the opposite shift in δ^{13}C and δ^{18}O values should be observed. Within the Legaire Sur burial population, we see a smaller shift of 0.4 ±0.1‰ in δ^{13}C and -0.3 ±0.1‰ between the M1 and M2 in all individuals–except for outlier SE12-19.405, which shows an opposite pattern (Table 3 and Fig 7). This may mean that the breastfeeding signal did not contribute greatly to the bulk δ^{13}C and δ^{18}O values acquired from the M1's crown enamel (representing ∼3 years of life), consistently with the early age of cessation of nursing suggested by incremental dentine isotope values and, perhaps, with an also early beginning of weaning. A greater variation is observed between the dM2 and the M1 in SE12-

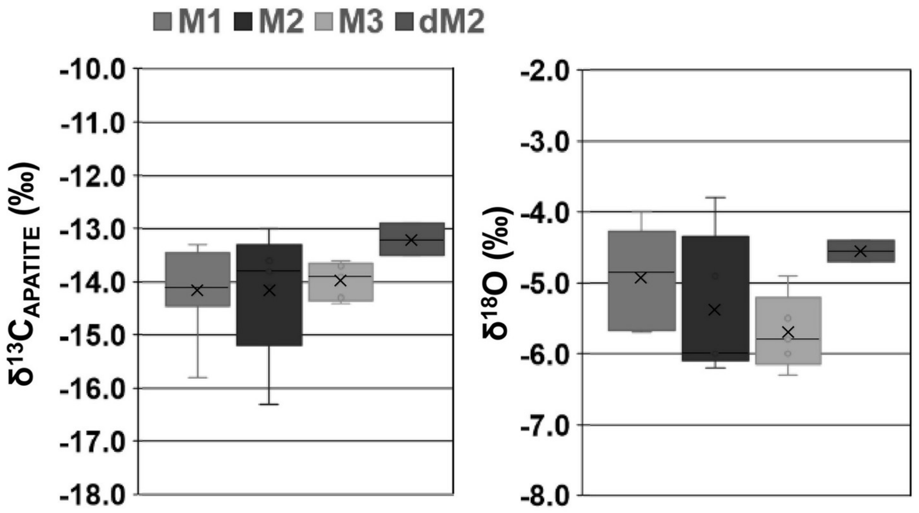

Fig 7. Range and median of δ^{13}Cap and δ^{18}OC values dependent on the tooth. (dm2 *n* = 2, M1 *n* = 8, M2 *n* = 6, M3 *n* = 6). In each plot, the X = average value, line = median value.

https://doi.org/10.1371/journal.pone.0316387.g007

20.1163, with a ^{13}C-depletion of -1.6‰, and an ^{18}O-depletion of -0.7‰. The dM2's crown enamel incorporates isotope values from 4 months *before* birth to <1 year after birth [70] (compared to the M1's crown enamel which forms *from* birth to ∼3 years old [42]). In theory, the enamel of the dM2 should provide a better representation of the breastfeeding period than the M1. For SE12-20.1163, the observed ^{13}C-depletion is consistent with the shift expected for the baby's transit from the mother's diet/values to breastfeeding. However, SE12-20.1163's ^{18}O-depletion is more difficult to address, since it cannot be interpreted as an early weaning. As such, for this individual, whilst a notable shift related to either diet or early life physiology can be observed, its cause is currently unidentifiable. Interestingly, SE12-20.758 (the only other individual with a sampled dM2), presents no notable depletion or increase in δ^{13}C and δ^{18}O values between their dM2 and the M1; a direct contrast with SE12-20.1163 values, meaning we cannot infer early weaning from this individual either.

The rationale for the rapid weaning observed in the study is unknown, although social-cultural reasons may be possible (e.g., [71]), particularly pastoralism, short-range residential mobility, and female labour. All of them fit well in the aforementioned Legaire scenario. Another consideration could be the prioritisation of nutrition towards the mother, especially if there were nutritional stresses within the population. However, there is no evidence for non-specific stress indicators in individuals from Legaire apart from a number of cases of *cribra crania* and linear enamel hypoplasia (LEH). Either way, early weaning (i.e., the complete cessation of breastfeeding before 2 years of age) appears to be a norm for the community buried at Legaire Sur.

Interestingly, the weaning strategy found at Legaire Sur is drastically different from the contemporaneous incremental dentine results found by [27] at Rioja Alavesa region. The average weaning age there is estimated between 3–4 years of age for individuals buried in both funerary caves and megalithic graves; with a slightly older average weaning age being observed in the megalithic tombs. The data from Legaire Sur megalithic grave indicates a notably different nursing strategy from the previously estimated for coeval regional groups, possibly related to different economic specialisations (mainly pastoral in Legaire Sur vs. mixed economies highly dependent on crop cultivation in megalithic groups of Rioja Alavesa region), potential mobility patterns (sedentarism vs. seasonal mobility) or possibly levels of social unrest. Whilst these rationales cannot yet be confirmed, it is interesting to observe that the evidence also compliments the aforementioned differences between the apatite isotope data from Legaire Sur and those in [27].

Reconstructing post-weaning diets through dentine collagen

The average post-weaning δ^{13}C and δ^{15}N of all individuals, aside from SE12-19.405, are closely clustered, indicating that these individuals likely all consumed a similar dietary source from a similar location. All individuals reveal a post-weaning diet with the plant contribution being from C_3 temperate environments [72], as expected in Iberia before the Bronze Age, where millets were introduced [28]. δ^{15}N values indicate the post-weaning consumption of terrestrial animal protein sources, which will be discussed in the following section (see: Adult diets) when compared to the faunal baseline. Individual SE12-19.405 shows a post-weaning δ^{13}C sequence which is notably lower than the other individuals (average = 1.5‰). Whilst she still has a similar diet to the rest of the population, this depletion provides further evidence (alongside the $\delta^{13}C_{ap}$, $\delta^{18}O_c$, $^{87}Sr/^{86}Sr$, and δ^{88}Sr taken from the enamel apatite) that this individual spent her childhood and adolescence consuming in a different geographical location to the rest of the burial population.

Although the visible averages of post-weaning $\delta^{15}N$ are similar across the burial population, there is some observable movement in the incremental patterns of some individuals. Whilst this could infer seasonal/age-related changes in diet, fluctuation in $\delta^{15}N$ values has also previously been attributed to physiological stresses, including nutritional stress, puberty, and pregnancy [73]. Across the $\delta^{15}N$ profiles of all sampled individuals, there is a clear ^{15}N-enrichment, which might be linked with either an early life dietary shift or potentially prolonged stress periods. For SE12-20.1288, after we observe a rapid weaning in the crown in the M1, the ^{15}N depletes, before immediately enriching again to a similar value as the breastfeeding signature (Fig 3). This pattern of immediate, post-weaning ^{15}N-enrichment (with a similar ^{13}C-enrichment being observed in the same increments) has previously been attributed to childhood nutritional stress and infant immune system complications [71], related this with the so-called "post-weanling's conundrum" (see [28] for a review). It may also be considered that whilst the individual had initially completely weaned, they were returned to breast milk soon after, so-called 'relactation'. The low Sr concentration in the M1 enamel could support this. Previous research has indicated that the consumption of breast milk results in low Sr concentrations, due to the high intake of calcium [67]. However, other environmental factors, such as consuming food from calcium-rich soils, may also be considered [74]. As the rest of the teeth are unavailable for this individual, it is impossible to determine if this comparatively higher $\delta^{15}N$ value continues post $\sim$12 years of age [16]. Unfortunately, the fragmentary and commingled nature of the skeletal assemblage prevents from addressing the real incidence of nutritional stress in the population, with the few osteological indicators that may be attributed to such deficiencies consisting of a number of cases of linear enamel hypoplasia in one maxilla and several isolated teeth, and of cribra crania in one skull and several isolated cranial fragments. This makes ^{15}N-enrichment difficult to fully attribute to nutritional stress alone, at least at the current state of the research.

Within the other individuals, ^{15}N-enrichment is observed in the roots of the M2 and within parts of the M3, representing a time of life of 13–18 years of age [35]. ^{15}N-enrichment at this age has previously been attributed to hormone-related changes linked with puberty [73] and is usually more apparent in women. Individuals SE12-19.405 and SE12-20.1334 are the only identified females with incrementally sampled M2s and/or M3s; both of which show notable ^{15}N-enrichment during this period. Whilst the effects of puberty may be one factor, others should be considered such as age-related changes in diet, with the gradual incorporation of higher trophic-level food [75], or at least the increased consumption of animal products with age at Legaire Sur. This may be exemplified on the M3 of SE-12-20.1373, which presents a $\sim$1‰ ^{13}C-depletion from 15-18/19 years of age, and a $\sim$1‰ ^{13}C-enrichment from 18/19-22 years of age. This indicates a consistent change in diet that occurs over several years, which may dietary intake changes from adolescence to adult life.

In this regard and at a general level, it is worth noting the progressive and marked increase in both $\delta^{13}C$ and $\delta^{15}N$ values observed in at least three individuals (e.g., SE12-19.405, SE12-20.1288, SE-12-20.1373) from the age of cessation of nursing (i.e., fully weaning) onwards. The interpretation of this pattern is difficult to address at present since it seems to be a consistent shift that transcends other minor, sinusoidal shifts potentially related to seasonal mobility, as suggested above. An explanation in dietary terms may be the rapid introduction and progressively greater consumption of high-trophic level foods, perhaps including dairy and/or meat products, after weaning, which may be in line with the hypothesis of a pastoralist way of life envisaged for the people living and burying their dead at Legaire.

Finally, the [Sr] data may also allude to further dietary changes, unobservable in the incremental dentine analysis. There is a notable diversity in values between individuals from Legaire Sur megalithic grave, with some individuals, such as SE12-20.1334, SE12-20.1373, and SE12-

20.486, showing notably high concentrations of Sr (159–185 ppm) in comparison to the rest of the population. These [Sr] are however relatively stable across the successionally forming teeth of most individuals, indicating little detectable dietary change, except for SE12-19.515. In this case, Sr concentration enrichment may suggest a shift in subsistence between the M1/M2 and the M3 (at some point between 5 and 9 years of age) [42] (Figs 1 and 2). This could indicate an age-related movement away from more calcium-rich food (excluding breast milk) [76]. Differences in [Sr] could also be attributed to the relative increase or decrease of meat and plant dietary contributions. Plant matter has more easily digestible strontium than that of meat, meaning it will contribute more strontium to forming body tissues [76, 77]. Inter and intra-individual [Sr] variation may be attributed to periods of a dietary focus more on either meat or plant. However, this dietary shift may also be due to movement around the local landscape, to a more calcium-depleted or strontium-enriched lithology, holding a similar bio-available $^{87}Sr/^{86}Sr$ [74].

Reconstructing juvenile and adult diets through bone collagen

The results of the bone collagen $\delta^{13}C$ and $\delta^{15}N$ analysis indicate that the average human diet is primarily composed of terrestrial protein sources from a C_3 temperate environment [72]; which has an expected mean $\delta^{13}C$ value for adult human bone collagen of ~-20.0‰ [78]. This is also seen in the human bone collagen data (average: $\delta^{13}C$: -20.1 ±0.5‰, $\delta^{15}N$: 8.6 ±0.4‰). There is minimal fractionation of $\delta^{13}C$ values through trophic levels (≤1.0‰) [79], although there is a substantial expected enrichment in $\delta^{15}N$ values of ~2.0‰ to 6.0‰, per trophic level [72, 80]. The $\delta^{13}C$ values closely align with the terrestrial livestock and a N^{15}-enrichment of ~4.0‰. This places our adult human diet at a trophic level above our faunal baseline, indicating that the average adult diet at Legaire Sur is probably primarily composed of regionally sourced, terrestrial livestock and plants. Whilst the majority of these individuals fit into this bracket, there are two individuals with $\delta^{15}N$ values that appear to be ~2.0‰ higher than the rest of the group (SE12-20.664: 10.6‰, SE12-20.758: 10.5‰). These individuals were classified as an infant (SE12-20.664, being estimated to be <1 year old), and as a young child (SE12-20.758 being ca. 3 years old). This is a frequent observation on the isotope composition of bone collagen of infants and early children [66, 81, 82], and is indicative of the consumption of breastmilk and/or physiological stress causing the ^{15}N-enrichment [83]. These values are not included when discussing the adolescent and adult dietary histories of the Legaire Sur population.

Interestingly, despite the notable provenance and weaning differences of individual SE12-19.405, her adult $\delta^{13}C$ and $\delta^{15}N$ bone values cluster closely with most of the other individuals, indicating that she may have consumed a similar diet to the rest of the community during her later adult life. This could indicate that this individual had fully integrated into this population.

Comparing the bone collagen data of Legaire Sur individuals to the broadly coeval Late Neolithic/Chalcolithic individuals analysed in [2, 4] ($n = 125$) in the Rioja Alavesa region (site $n = 8$) (Fig 8), reveals 'similar' terrestrial protein, C_3 plant-based diets. However, there is a slight N^{15}-enrichment among the individuals from caves and megalithic graves from the Rioja Alavesa region (megalithic graves average: 9.6‰, funerary cave average: 9.4‰) in comparison to those found in the bone collagen from the Legaire Sur megalithic grave (average: 8.8‰). Whilst this ^{15}N-enrichment is insignificant to qualify dietary types, it provides further supportive evidence to the $\delta^{18}O$ isotope and [Sr] data, that the individuals buried at Legaire Sur were likely consuming resources in a different geographic location than the individuals buried at the other megalithic graves to the south of the site (distance > 35 km) [29] analysed the bone collagen of 22 humans from Pico Ramos (~88km away from Legaire Sur), finding a similar

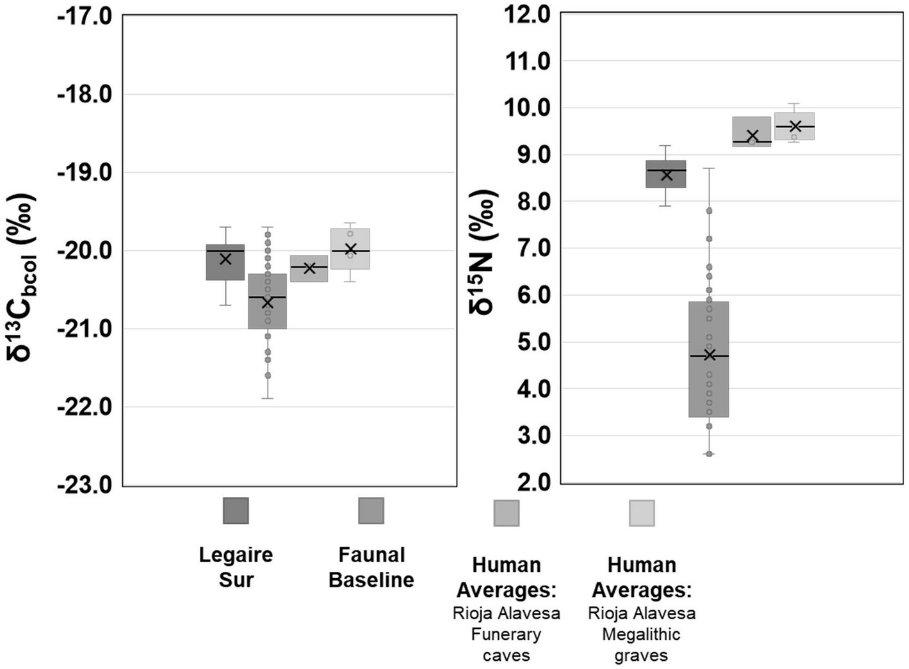

Fig 8. δ^{13}C and δ^{15}N values from adult human bone collagen of Legaire Sur plotted with the collated values of the faunal baseline (Fernandez-Crespo and Schulting 2017 and Sarasketa-Gartzia *et al.* 2018), and the average site values of the Rioja Alavesa funerary caves (Las Yurdinas II, Los Husos I, Peña Larga) and megalithic graves (El Sotillo, San Martín, Longar, Chabola de la Hechicera, Alto de la Huesera) (collated from: [2, 4]). In each plot, the X = average value, line = median value.

https://doi.org/10.1371/journal.pone.0316387.g008

δ^{13}C$_{bcol}$ range of −21.0‰ to −19.9‰ and a δ^{15}N$_{bcol}$ value range of 8.2‰ to 9.8‰. Despite this site being far closer to the coast, the authors conclude similarly, determining that the primary components within the adult diets of these Late Neolithic/Chalcolithic individuals originated from C_3 terrestrial resources.

In many regions of Iberia, it has been suggested that Neolithic subsistence may have relied on animal husbandry together with different degrees of agriculture, hunting and gathering (e.g., [84]). In the case of Legaire Sur, Arakil valley lowland pastures and fall and winter nuts, fruits, fungi and green vegetables gathering would support both flocks and people, and complement whatever contribution a small-scale crop and perhaps legume cultivation could have provided during the cold months. Subsistence in spring and summer time in the uplands may have relied on stored cultivated and wild plants obtained from the lowlands together with the potential exploitation of milk and dairy products and some eventual wild game such as hare, red deer or wild boar [2].

Conclusion

Combining isotope data with sampling incremental tooth dentine, bulk tooth enamel, and bulk bone collagen has enabled the intra-individual reconstruction of human behaviour regarding mobility and diet in Legaire Sur passage tomb. There, incremental dentine analyses reveal that weaning ends quite early, before two years in most cases. Post-weaning biographies reveal specific childhood/adolescent diets; based upon terrestrial protein sources, with a C_3 plant contribution. In adolescence, there is evidence for distinct diets, with the potential increased consumption of higher trophic level animals, although metabolic processes, such as puberty or physiological stress, may have been a cause. The diet's primary animal protein

intake component remains terrestrial, a trend that continues into adult life, and is reflected in the sampled bone collagen.

Comparing the $^{87}Sr/^{86}Sr$ ratios from the human tooth enamel with the available geographical baseline indicates that all the individuals could have lived within a ∼35km radius of the site. Consistently, the results from the apatite clearly show a close clustering of individuals, likely indicating they lived and consumed food from the same landscapes. All except SE12-19.405 (female), who despite still holding values that may be considered 'regional', clearly had a childhood in a different geological location. This is supported by the $\delta^{13}C_{ap}$, $\delta^{88}Sr$, and incremental $\delta^{13}C_{dcol}$ values, which indicate that, whilst this individual had a similar dietary composition, she likely exploited a different environment. It is only in adult life, when comparing the bone collagen data, that this individual's diet becomes more closely aligned with the rest of the community.

When compared to the previous work of Fernández-Crespo *et al.* (2020) on coeval individuals from funerary caves and megalithic graves, notable differences in lifeways are observed. The people of Legaire Sur held different nursing strategies and had slightly different adult diets (low valley vs. upland environmental exploitation). Fernández-Crespo *et al.* (2020) concluded a "them and us" scenario between funerary cave communities and megalithic grave communities, which inhabited the same environment but had different lifeways, utilising and occupying the landscape in different ways. The reconstructed lifeways of those inhumed at Legaire Sur do not fit with those found in the funerary caves of Rioja Alavesa region, but neither with those found in the megalithic graves. Therefore, at Legaire Sur a separate population in the Basque region may be observed, one whose lifeways have been adapted seasonally to a hostile subalpine environment. The notable difference in the environment likely resulted in different subsistence and survival strategies to be employed, compared to those contemporaneously living in the lower Rioja Alavesa landscapes. Ultimately, whilst it has previously been hypothesised that social differences and cultural changes may have largely influenced lifeways in the Late Neolithic/Chalcolithic Basque country, the Legaire Sur population reveals the substantial influence that the environment also held on the lifeways in the region.

Supporting information

S1 File. Collagen extraction & faunal baseline.
(DOCX)

S2 File. Inclusivity in global research.
(DOCX)

Acknowledgments

The authors would also like to thank David Verstraeten, Wim Borremans, and Martyna Kopeć for their technical support, and Sam Hermans for their help with editing and design of the figures.

Author Contributions

Conceptualization: Jacob I. Griffith, Javier Ordoño, Barbara Veselka, Christophe Snoeck.

Data curation: Jacob I. Griffith, Hannah F. James, Rachèl Spros, Christophe Snoeck.

Formal analysis: Jacob I. Griffith, Hannah F. James, Javier Ordoño, Carina T. Gerritzen, Christophe Snoeck.

Funding acquisition: Jacob I. Griffith, Philippe Claeys, Steven Goderis, Christophe Snoeck.

Investigation: Jacob I. Griffith, Christophe Snoeck.

Methodology: Jacob I. Griffith, Teresa Fernández-Crespo, Carina T. Gerritzen, Christina Cheung, Christophe Snoeck.

Project administration: Christophe Snoeck.

Resources: Javier Ordoño, Philippe Claeys, Steven Goderis, Christophe Snoeck.

Supervision: Barbara Veselka, Christophe Snoeck.

Validation: Christina Cheung, Rachèl Spros.

Visualization: Jacob I. Griffith, Hannah F. James, Javier Ordoño, Teresa Fernández-Crespo.

Writing – original draft: Jacob I. Griffith, Javier Ordoño, Christophe Snoeck.

Writing – review & editing: Jacob I. Griffith, Hannah F. James, Javier Ordoño, Teresa Fernández-Crespo, Carina T. Gerritzen, Christina Cheung, Rachèl Spros, Philippe Claeys, Steven Goderis, Barbara Veselka, Christophe Snoeck.

References

1. Lillios KT. The Archaeology of the Iberian Peninsula: From the Paleolithic to the Bronze Age. Cambridge University Press; 2019.
2. Fernández-Crespo T, Ordoño J, Schulting RJ. Subsistence shift and socio-economic response to cultural and climate changes among north-central Iberian megalithic groups. Journal of Archaeological Science. 2021 1; 133:105451.
3. Weiss-Krejci E. Shedding Light on Dark Places. Deposition of the Dead in Caves and Cave-Like Features in Neolithic and Copper Age Iberia. Caves in Context. The Cultural Significance of Caves and Rockshelters in Europe. Oxbow. Oxford. 2012:118–37.
4. Fernández-Crespo T, Schulting RJ. Living different lives: early social differentiation identified through linking mortuary and isotopic variability in Late Neolithic/Early Chalcolithic north-central Spain. PLoS One. 2017 27; 12(9):e0177881. https://doi.org/10.1371/journal.pone.0177881 PMID: 28953890
5. Armendariz J, Irigarai S, Etxeberria F. New evidence of prehistoric arrow wounds in the Iberian Peninsula. International Journal of Osteoarchaeology. 1994; 4(3):215–22.
6. Fernández-Crespo T. An arrowhead injury in a Late Neolithic/Early Chalcolithic human cuneiform from the rockshelter of La Peña de Marañón (Navarre, Spain). International Journal of Osteoarchaeology. 2016; 26(6):1024–33.
7. Fernández-Crespo T. New evidence of early chalcolithic interpersonal violence in the Middle Ebro Valley (Spain): two arrowhead injuries from the swallet of Las Yurdinas II. International Journal of Osteoarchaeology. 2017; 27(1):76–85.
8. Fernández-Crespo T, Ordoño J, Etxeberria F, Herrasti L, Armendariz Á, Vegas JI, et al. Large-scale violence in Late Neolithic Western Europe based on expanded skeletal evidence from San Juan ante Portam Latinam. Scientific Reports. 2023 2; 13(1):17103. https://doi.org/10.1038/s41598-023-43026-9 PMID: 37919365
9. Gerritzen CT, Goderis S, James HF, Snoeck C. Optimizing Zr-doped MC-ICP-MS sample-standard bracketing to simultaneously determine 87Sr/86Sr and δ88Sr for high sample-throughput. Spectrochimica Acta Part B: Atomic Spectroscopy. 2024 26:106955.
10. Guiserix D, Albalat E, Ueckermann H, Davechand P, Iaccheri LM, Bybee G, et al. Simultaneous analysis of stable and radiogenic strontium isotopes in reference materials, plants and modern tooth enamel. Chemical Geology. 2022 20; 606:121000.
11. Lewis J, Pike AW, Coath CD, Evershed RP. Strontium concentration, radiogenic (87Sr/86Sr) and stable (δ88Sr) strontium isotope systematics in a controlled feeding study. STAR: Science & Technology of Archaeological Research. 2017 1; 3(1):45–57.
12. Reynard LM, Henderson GM, Hedges RE. Calcium isotope ratios in animal and human bone. Geochimica et Cosmochimica Acta. 2010 1; 74(13):3735–50.
13. McCormack J, Szpak P, Bourgon N, Richards M, Hyland C, Méjean P, et al. Zinc isotopes from archaeological bones provide reliable trophic level information for marine mammals. Communications biology. 2021 3; 4(1):683.

14. Beaumont J, Gledhill A, Montgomery J. Isotope analysis of incremental human dentine: towards higher temporal resolution. Bulletin of the International Association for Paleodontology. 2014 17; 8(2):212–23.

15. Cheung C, Fernández-Crespo T, Mion L, Di Giusto M, Goude G, Macdonald RA, et al. Micro-punches versus micro-slices for serial sampling of human dentine: Striking a balance between improved temporal resolution and measuring additional isotope systems. Rapid Communications in Mass Spectrometry. 2022 15; 36(21):e9380. https://doi.org/10.1002/rcm.9380 PMID: 35986908

16. Czermak A, Fernández-Crespo T, Ditchfield PW, Lee-Thorp JA. A guide for an anatomically sensitive dentine microsampling and age-alignment approach for human teeth isotopic sequences. American Journal of Physical Anthropology. 2020; 173(4):776–83. https://doi.org/10.1002/ajpa.24126 PMID: 32779777

17. Cheung C, Szpak P. Interpreting past human diets using stable isotope mixing models—best practices for data acquisition. Journal of Archaeological Method and Theory. 2022; 29(1):138–61.

18. Fernandes R, Millard AR, Brabec M, Nadeau MJ, Grootes P. Food reconstruction using isotopic transferred signals (FRUITS): a Bayesian model for diet reconstruction. PloS one. 2014 13; 9(2):e87436. https://doi.org/10.1371/journal.pone.0087436 PMID: 24551057

19. Price TD. An introduction to the isotopic studies of ancient human remains. Journal of the North Atlantic. 2014;2014(sp7):71–87.

20. DeNiro MJ. You are what you eat (plus a few‰): the carbon isotope cycle in food chains. Ingeological society of America abstracts with programs 1976 (Vol. 8, p. 834).

21. Richards MP, Schulting RJ, Hedges RE. Sharp shift in diet at onset of Neolithic. Nature. 2003 25; 425 (6956):366.

22. Slovak NM, Paytan A. Applications of Sr isotopes in archaeology. Handbook of Environmental Isotope Geochemistry: Vol I. 2012:743–68.

23. Dalle S, Snoeck C, Sengeløv A, Salesse K, Hlad M, Annaert R, et al. Strontium isotopes and concentrations in cremated bones suggest an increased salt consumption in Gallo-Roman diet. Scientific reports. 2022 3; 12(1):9280. https://doi.org/10.1038/s41598-022-12880-4 PMID: 35660749

24. Guiserix D, Dodat PJ, Jaouen K, Albalat E, Cardoso JM, Maureille B, et al. Stable isotope composition and concentration systematics of Ca and trace elements (Zn, Sr) in single aliquots of fossil bone and enamel. Geochimica et Cosmochimica Acta. 2024 15; 367:123–32.

25. de Winter NJ, Snoeck C, Schulting R, Fernández-Crespo T, Claeys P. High-resolution trace element distributions and models of trace element diffusion in enamel of Late Neolithic/Early Chalcolithic human molars from the Rioja Alavesa region (north-central Spain) help to separate biogenic from diagenetic trends. Palaeogeography, Palaeoclimatology, Palaeoecology. 2019 15; 532:109260.

26. Fernández-Crespo T, Czermak A, Lee-Thorp JA, Schulting RJ. Infant and childhood diet at the passage tomb of Alto de la Huesera (north-central Iberia) from bone collagen and sequential dentine isotope composition. International Journal of Osteoarchaeology. 2018; 28(5):542–51.

27. Fernández-Crespo T, Snoeck C, Ordoño J, De Winter NJ, Czermak A, Mattielli N, et al. Multi-isotope evidence for the emergence of cultural alterity in Late Neolithic Europe. Science Advances. 2020 22;6 (4):eaay2169. https://doi.org/10.1126/sciadv.aay2169 PMID: 32010785

28. Fernández-Crespo T, Schulting RJ, Czermak A, Ordoño J, Lorenzo JI, Rodanés JM. The "post-weanling's conundrum": exploring the impact of infant and child feeding practices on early mortality in the Bronze Age burial cave of Moro de Alins, north-eastern Iberia, through stable isotope analysis. Archaeological and Anthropological Sciences. 2022; 14(10):196.

29. Sarasketa-Gartzia I, Villalba-Mouco V, Le Roux P, Arrizabalaga Á, Salazar-García DC. Late Neolithic-Chalcolithic socio-economical dynamics in northern Iberia. A multi-isotope study on diet and provenance from Santimamiñe and Pico Ramos archaeological sites (Basque Country, Spain). Quaternary International. 2018 10; 481:14–27.

30. Dean MC, Scandrett AE. Rates of dentine mineralization in permanent human teeth. International Journal of Osteoarchaeology. 1995; 5(4):349–58.

31. Maestu IB, Aramburu JI. Los grupos humanos en la prehistoria de Encia y Urbasa. Análisis cultural de asentamientos, sistemas de explotación, modos de vida y ritos desde el neolítico hasta el final de la edad antigua: síntesis final. 1990.

32. Eguren E. Los dólmenes clásicos alaveses: nuevos dólmenes en la Sierra de Entzia (Encía). Revista internacional de los estudios vascos = Eusko ikaskuntzen nazioarteko aldizkaria = Revue internationale des ètudes basques = International journal on Basque studies, RIEV. 1927; 18(1):1–54.

33. Ordoño J, Martínez LM, Alday A, Niso J; Loza M; Fernández-Crespo T. Parque Megalítico de Legaire (Araba/Álava). II campaña. Arkeoikuska: Investigación 2019. 2020: 225–235.

34. Ordoño J, Martínez LM, Alday A, Niso J, Loza M, Fernández-Crespo T. Parque Megalítico de Legaire (Araba/Álava). III campaña: dolmen de Legaire Sur. Arkeoikuska: Investigación 2020. 2021: 105–112.

35. AlQahtani SJ, Hector MP, Liversidge HM. Brief communication: The London atlas of human tooth development and eruption. American Journal of physical anthropology. 2010; 142(3):481–90. https://doi.org/10.1002/ajpa.21258 PMID: 20310064

36. Buikstra JE. Standards for data collection from human skeletal remains. Arkansas archaeological survey research series. 1994; 44:18.

37. Brothwell DR. Digging up bones: the excavation, treatment, and study of human skeletal remains. Cornell University Press; 1981.

38. Hanáková H. A Brief Outline of the Anthropological Research of the Bronze Age in Bohemia. Anthropologie (1962-). 1978 Jan 1:111–6.

39. Scheuer L, Black S. Development and ageing of the juvenile skeleton. Human osteology in archaeology and forensic science. 2000 Jan 2:9–22.

40. Ferembach D. Recomendations for age and sex diagnosis of skeletons. Journal of human evolution. 1980; 9:517–49.

41. Zambrano CJ. Evaluation of regression equations used to estimate age at death from cranial suture closure (Doctoral dissertation, University of Indianapolis).

42. Reid DJ, Dean MC. Variation in modern human enamel formation times. Journal of human evolution. 2006 1; 50(3):329–46. https://doi.org/10.1016/j.jhevol.2005.09.003 PMID: 16300817

43. Plomp E, von Holstein IC, Kootker LM, Verdegaal-Warmerdam SJ, Forouzanfar T, Davies GR. Strontium, oxygen, and carbon isotope variation in modern human dental enamel. American journal of physical anthropology. 2020; 172(4):586–604. https://doi.org/10.1002/ajpa.24059 PMID: 32333689

44. Wright LE. Examining childhood diets at Kaminaljuyu, Guatemala, through stable isotopic analysis of sequential enamel microsamples. Archaeometry. 2013 Feb; 55(1):113–33.

45. Snoeck C, Pellegrini M. Comparing bioapatite carbonate pre-treatments for isotopic measurements: Part 1—Impact on structure and chemical composition. Chemical Geology. 2015 6; 417:394–403.

46. Pellegrini M, Snoeck C. Comparing bioapatite carbonate pre-treatments for isotopic measurements: Part 2—Impact on carbon and oxygen isotope compositions. Chemical Geology. 2016 20; 420:88–96.

47. De Winter NJ, Snoeck C, Claeys P. Seasonal cyclicity in trace elements and stable isotopes of modern horse enamel. PLoS One. 2016 22; 11(11):e0166678. https://doi.org/10.1371/journal.pone.0166678 PMID: 27875538

48. Balcaen L, Bolea-Fernandez E, Resano M, Vanhaecke F. Inductively coupled plasma–Tandem mass spectrometry (ICP-MS/MS): A powerful and universal tool for the interference-free determination of (ultra) trace elements–A tutorial review. Analytica chimica acta. 2015 24; 894:7–19.

49. Snoeck C, Lee-Thorp J, Schulting R, De Jong J, Debouge W, Mattielli N. Calcined bone provides a reliable substrate for strontium isotope ratios as shown by an enrichment experiment. Rapid communications in mass spectrometry. 2015 15; 29(1):107–14. https://doi.org/10.1002/rcm.7078 PMID: 25462370

50. De Laeter JR, Böhlke JK, De Bievre P, Hidaka H, Peiser HS, Rosman KJ, et al. Atomic weights of the elements. Review 2000 (IUPAC Technical Report). Pure and applied chemistry. 2003 1; 75(6):683–800.

51. Weis D, Kieffer B, Maerschalk C, Barling J, De Jong J, Williams GA, et al. High-precision isotopic characterization of USGS reference materials by TIMS and MC-ICP-MS. Geochemistry, Geophysics, Geosystems. 2006;7(8).

52. Lazzerini N, Balter V, Coulon A, Tacail T, Marchina C, Lemoine M, et al. Monthly mobility inferred from isoscapes and laser ablation strontium isotope ratios in caprine tooth enamel. Scientific Reports. 2021 26; 11(1):2277. https://doi.org/10.1038/s41598-021-81923-z PMID: 33500495

53. Dean MC, Scandrett AE. The relation between long-period incremental markings in dentine and daily cross-striations in enamel in human teeth. Archives of Oral Biology. 1996 1; 41(3):233–41. https://doi.org/10.1016/0003-9969(95)00137-9 PMID: 8735009

54. Kawasaki K, Tanaka S, Ishikawa T. On the daily incremental lines in human dentine. Archives of oral biology. 1979 1; 24(12):939–43. https://doi.org/10.1016/0003-9969(79)90221-8 PMID: 232978

55. DeNiro MJ. Postmortem preservation and alteration of in vivo bone collagen isotope ratios in relation to palaeodietary reconstruction. nature. 1985 31; 317(6040):806–9.

56. Wilson T, Szpak P. Comparing the performance of demineralization agents (HCl and EDTA) for stable isotope analysis of bone collagen with implications for quality control criteria and collagen yield. International Journal of Osteoarchaeology. 2023; 33(3):506–16.

57. Robador Moreno A, Samsó Escolá JM, Ramajo Cordero J, Barnolas Cortinas A., Clariana García P., Martín Alfageme S y Gil Peña, I. Mapa Geológico Digital continuo E. 1: 50.000, Zona Pirineos Vasco-Cantábrica (Zona-1600). in GEODE. Mapa Geológico Digital continuo de España.[en línea]. [Fecha de consulta]. Disponible en: http://info.igme.es/cartografiadigital/geologica/geodezona.aspx?Id=Z1600

58. Runfola D, Anderson A, Baier H, Crittenden M, Dowker E, Fuhrig S, et al. geoBoundaries: A global database of political administrative boundaries. PloS one. 2020 Apr 24; 15(4):e0231866. https://doi.org/10.1371/journal.pone.0231866 PMID: 32330167
59. Schwarcz HP, Schoeninger MJ. Stable isotope analyses in human nutritional ecology. American Journal of Physical Anthropology. 1991; 34(S13):283–321.
60. Berto D, Rampazzo F, Gion C, Noventa S, Formalewicz M, Ronchi F, et al. Elemental analyzer/isotope ratio mass spectrometry (EA/IRMS) as a tool to characterize plastic polymers in a marine environment. Plastics in the environment'.(Ed. A Gomiero) pp. 2019 21:37–54.
61. Knipper C, Mittnik A, Massy K, Kociumaka C, Kucukkalipci I, Maus M, et al. Female exogamy and gene pool diversification at the transition from the Final Neolithic to the Early Bronze Age in central Europe. Proceedings of the National Academy of Sciences. 2017 Sep 19; 114(38):10083–8.
62. Lightfoot E, O'Connell TC. On the use of biomineral oxygen isotope data to identify human migrants in the archaeological record: intra-sample variation, statistical methods and geographical considerations. PloS one. 2016 28; 11(4):e0153850. https://doi.org/10.1371/journal.pone.0153850 PMID: 27124001
63. Barandiaran J.M, Manterola A (Eds). 2000. Atlas etnográfico de Vasconia. Ganadería y pastoreo en Vasconia. Eusko Jaurlaritza, Gobierno de Navarra, Etniker Euskalerria.
64. Fogel ML, Tuross N, Owsley DW. Nitrogen isotope tracers of human lactation in modern and archaeological populations. Carnegie Inst. Washington Yearbook. 1989; 88: 111–117.
65. Fuller BT, Fuller JL, Harris DA, Hedges RE. Detection of breastfeeding and weaning in modern human infants with carbon and nitrogen stable isotope ratios. American Journal of Physical Anthropology: The Official Publication of the American Association of Physical Anthropologists. 2006; 129(2):279–93. https://doi.org/10.1002/ajpa.20249 PMID: 16261548
66. Breastfeeding Jay M. and weaning behaviour in archaeological populations: evidence from the isotopic analysis of skeletal materials. Childhood in the Past. 2009 1; 2(1):163–78.
67. Tsutaya T, Yoneda M. Reconstruction of breastfeeding and weaning practices using stable isotope and trace element analyses: A review. American journal of physical anthropology. 2015; 156:2–1. https://doi.org/10.1002/ajpa.22657 PMID: 25407359
68. Chinique de Armas Y, Mavridou AM, Garcell Domínguez J, Hanson K, Laffoon J. Tracking breastfeeding and weaning practices in ancient populations by combining carbon, nitrogen and oxygen stable isotopes from multiple non-adult tissues. PloS one. 2022 2; 17(2):e0262435. https://doi.org/10.1371/journal.pone.0262435 PMID: 35108296
69. Wright LE, Schwarcz HP. Stable carbon and oxygen isotopes in human tooth enamel: identifying breastfeeding and weaning in prehistory. American Journal of Physical Anthropology: The Official Publication of the American Association of Physical Anthropologists. 1998; 106(1):1–8.
70. Brich W, Dean MC. A new method of calculating human deciduous enamel formation times. Bulletin of the International association for paleodontology. 2013 Jun 29; 7(1):47–55.
71. Henderson RC, Lee-Thorp J, Loe L. Early life histories of the London poor using δ13C and δ15N stable isotope incremental dentine sampling. American Journal of Physical Anthropology. 2014; 154(4):585–93.
72. Lee-Thorp JA. On isotopes and old bones. Archaeometry. 2008; 50(6):925–50.
73. D'Ortenzio L, Brickley M, Schwarcz H, Prowse T. You are not what you eat during physiological stress: Isotopic evaluation of human hair. American journal of physical anthropology. 2015; 157(3):374–88. https://doi.org/10.1002/ajpa.22722 PMID: 25711625
74. Evans JA, Chenery CA, Montgomery J. A summary of strontium and oxygen isotope variation in archaeological human tooth enamel excavated from Britain. Journal of Analytical Atomic Spectrometry. 2012; 27(5):754–64.
75. Avery LC, Brickley MB, Findlay S, Chapelain de Seréville-Niel C, Prowse TL. Child and adolescent diet in Late Roman Gaul: An investigation of incremental dietary stable isotopes in tooth dentine. International journal of Osteoarchaeology. 2021; 31(6):1226–36.
76. Lahtinen M, Arppe L, Nowell G. Source of strontium in archaeological mobility studies—marine diet contribution to the isotopic composition. Archaeological and Anthropological Sciences. 2021; 13:1–0.
77. Montgomery J. Passports from the past: Investigating human dispersals using strontium isotope analysis of tooth enamel. Annals of human biology. 2010 1; 37(3):325–46. https://doi.org/10.3109/03014461003649297 PMID: 20367186
78. Smith BN, Epstein S. Two categories of 13C/12C ratios for higher plants. Plant physiology. 191; 47 (3):380–4.
79. Codron D, Clauss M, Codron J, Tütken T. Within trophic level shifts in collagen–carbonate stable carbon isotope spacing are propagated by diet and digestive physiology in large mammal herbivores. Ecology and evolution. 2018; 8(8):3983–95. https://doi.org/10.1002/ece3.3786 PMID: 29721273

80. Sealy JC, Van Der Merwe NJ, Thorp JA, Lanham JL. Nitrogen isotopic ecology in southern Africa: implications for environmental and dietary tracing. Geochimica et Cosmochimica Acta. 1987 1; 51 (10):2707–17.

81. Richards MP, Mays S, Fuller BT. Stable carbon and nitrogen isotope values of bone and teeth reflect weaning age at the Medieval Wharram Percy site, Yorkshire, UK. American Journal of Physical Anthropology: The Official Publication of the American Association of Physical Anthropologists. 2002; 119 (3):205–10. https://doi.org/10.1002/ajpa.10124 PMID: 12365032

82. Gardner KS, Bartelink EJ, Martinez A, Leventhal A, Cambra R. Breastfeeding and weaning practices of the ancestral Ohlone Indians of California: A case study using stable isotope analysis of bone collagen. International Journal of Osteoarchaeology. 2018; 28(5):523–34.

83. Beaumont J, Atkins EC, Buckberry J, Haydock H, Horne P, Howcroft R, et al. Comparing apples and oranges: Why infant bone collagen may not reflect dietary intake in the same way as dentine collagen. American Journal of Physical Anthropology. 2018; 167(3):524–40. https://doi.org/10.1002/ajpa.23682 PMID: 30187451

84. Cruz AR, Cuenca EC, editors. Rendering Death: Ideological and Archaeological Narratives from Recent Prehistory Iberia. Archaeopress; 2014.
